# PARylation of HMGA1 desensitizes esophageal squamous cell carcinoma to olaparib

**DOI:** 10.1002/ctm2.70111

**Published:** 2024-12-17

**Authors:** Xin‐Yuan Lei, Kai‐Yue He, Qiu‐Tong Li, Lei Zhang, Dan‐Hui Wu, Jing‐Yu Yang, Jin‐Rong Guo, Meng‐Jie Liu, Zi‐Long Zhao, Jun‐Qi Li, Huai Liu, Yuan Zhao, Yu‐Jia Li, Qian‐Hui Sun, Chen‐Guang Wu, Yun‐Fan Wang, Geng‐Sheng Cao, Gang Wang, Yong‐Ping Jian, Zhi‐Xiang Xu

**Affiliations:** ^1^ School of Life Sciences Henan University Kaifeng China

**Keywords:** chemotherapy sensitivity, DNA damage, ESCC, HMGA1, Olaparib, PARP1, PARylation

## Abstract

As a chromatin remodelling factor, high mobility group A1 (HMGA1) plays various roles in both physiological and pathological conditions. However, its role in DNA damage response and DNA damage‐based chemotherapy remains largely unexplored. In this study, we report the poly ADP‐ribosylation (PARylation) of HMGA1 during DNA damage, leading to desensitization of esophageal squamous cell carcinoma (ESCC) cells to the poly(ADP‐ribose) polymerase 1 (PARP1) inhibitor, olaparib. We found that HMGA1 accumulates at sites of DNA damage, where it interacts with PARP1 and undergoes PARylation at residues E47 and E50 in its conserved AT‐hook domain. This modification enhances the accumulation of Ku70/Ku80 at the site of DNA damage and activates the DNA‐dependent protein kinase catalytic subunit, facilitating nonhomologous end‐joining repair. In both subcutaneous tumour models and genetically engineered mouse models of in situ esophageal cancer, HMGA1 interference increased tumour sensitivity to olaparib. Moreover, HMGA1 was highly expressed in ESCC tissues and positively correlated with PARP1 levels as well as poor prognosis in ESCC patients. Taken together, these findings reveal a mechanistic link between HMGA1 and PARP1 in regulating cell responses to DNA damage and suggest that targeting HMGA1 could be a promising strategy to increase cancer cell sensitivity to olaparib.

## Graphical abstract

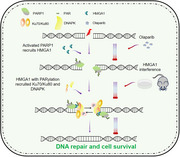

## 1 INTRODUCTION

Esophageal cancer (EC) is one of the most common malignant tumours in the world, with high morbidity and mortality, which seriously affects public health.^1^, ^2^ There are two primary histological types of EC: ESCC and esophageal adenocarcinoma (EAC).^3^, ^4^ ESCC is the most common form and accounts for the highest incidence and mortality rates, particularly in Eastern countries. Overall, EC ranks seventh in incidence and sixth in mortality.^5^

Similar to other cancers, genome instability is a key driver of ESCC.^6^, ^7^ Timely repair of DNA damage is essential for maintaining genomic stability. Nonhomologous end join (NHEJ) and homologous recombination (HR) are two important ways to repair DNA double‐strand breaks (DSBs), and among the many forms of DNA damage, DSBs are the most severe form.^8^, ^9^, ^10^ In ESCC, the predominant repair pathway is canonical NHEJ (cNHEJ), which needs minimal DNA terminal processing. This process is activated by the binding of the XRCC5/XRCC6(Ku80/Ku70) heterodimer to the broken DNA ends, which activates the DNA‐dependent protein kinase catalytic subunit (DNA‐PKcs).^11^ Defects in these pathways lead to increased mutations and chromosome rearrangements, potentially driving genome instability and enhancing cancer progression.^12^ On the other hand, overactivation of DSB repair patterns such as NHEJ and HR may support tumour maintenance by helping proliferating cancer cells cope with high levels of DNA damage induced by replication stress and oxidative stress.^13^, ^14^

PARPs are a family of proteins that transfer ADP‐ribose groups to target proteins, playing an important role in DNA damage repair and genome stabilization.^15^, ^16^ Poly(ADP‐ribose) polymerase 1 (PARP1), the central enzyme responsible for poly(ADP‐ribosyl)ation in response to DNA damage, is key to the DNA damage response.^16^ PARylation contributes to DNA repair processes such as base excision repair (BER), single‐strand break repair, nucleotide excision repair (NER), and double‐strand break repair.^17^, ^18^

PARP1 is activated by binding to broken DNA and accelerates chromatin expansion by modifying neighbouring nucleoproteins such as histones. This process facilitates the recruitment of ATP‐dependent chromatin remodelers near the DNA gaps.^19^ PARP1 activation increases the accessibility of chromatin, promoting the accumulation of DSB repair proteins such as XRCC4, Ku70 and Ku80.^20^, ^21^ ZNF384, a transcription factor containing a zinc finger (ZnF) domain, has been shown to be accumulated to the site of DNA damage via PARP1‐mediated chromatin expansion, promoting cNHEJ through its N‐terminal interaction with Ku70/Ku80.^22^

PARP inhibitors (PARPis) have shown significant success in treating cancers with homologous recombination (HR) deficiencies.^23^ Olaparib is a PARP inhibitor that has been approved for maintenance therapy after first‐line chemotherapy in advanced ovarian cancer.^24^, ^25^ However, like other cytotoxic drugs, resistance to PARPis, including olaparib, remains a significant challenge.^26^, ^27^ While olaparib has begun to be used in EC treatment, its therapeutic potential is still being explored, with some studies suggesting that olaparib may enhance the efficacy of other anticancer drugs in EC.^28^, ^29^, ^30^, ^31^

HMGA1 is a nonhistone chromatin‐binding protein expressed in embryonic tissues and embryonic stem cells, but largely absent in normal adult somatic cells.^32^, ^33^, ^34^ However, HMGA1 is re‐expressed in cancer cells and cancer stem cells, where it is associated with enhanced metastasis and poor clinical prognosis.^35^, ^36^ HMGA1 binds AT‐rich DNA sequences via its three AT‐hook domains, causing DNA conformational changes that modulate transcription.^33^, ^37^ Under conditions of genetic stress, HMGA1 promotes DNA BER, increasing cellular survival.^37^ This property renders HMGA1‐positive cells more resistant to therapies targeting DNA base damage.^38^ HMGA1 has also been implicated in DNA damage repair and telomere stability in Arabidopsis thaliana.^39^ Its role in BER is vital for maintaining genome stability and early embryonic development.^40^

Previous research has shown that increased HMGA1 expression in ESCC promotes tumorigenesis and that HMGA1 downregulation inhibits pentose phosphate pathway (PPP) by suppressing recombinant transketolase (TKT) transcription.^31^, ^41^ Additionally, HMGA1 has been shown to enhance resistance to cisplatin in ESCC cells.^42^ To elucidate the role of HMGA1 in DNA repair and the development of EC, we conducted experiments focused on HMGA1 and its role in DNA damage repair. In this study, we discovered that HMGA1 undergoes PARylation in response to DNA damage, which promotes Ku protein recruitment and activates DNA‐PKcs, thereby enhancing DNA repair and reducing cancer cell sensitivity to the PARP inhibitor olaparib. We further found that HMGA1 depletion sensitizes ESCC to olaparib, suggesting a potential therapeutic target for enhancing cancer treatment efficacy.

## 2 MATERIALS AND METHODS

### 2.1 Antibodies and chemicals

The antibody against Ki67 was from Abcam. The antibodies against HMGA1, PARP1, Ku70, Ku80, γ‐H2AX, DNA‐PKcs, histone H3, and dsDNA markers were from Santa Cruz Biotechnology. Poly/mono‐ADP ribose (E6F6A) and p‐DNA‐PKcs were sourced from Cell Signaling Technology. GAPDH, β‐actin, anti‐HA, anti‐DYKDDDDK‐tag, and anti‐GFP were sourced from Protein Technology. Etoposide, olaparib, and gallotannin were procured from Meilun Bio, and AZD5305 and PDD0017273 were purchased from MCE.

### 2.2 Cell culture and treatment

The HEK293, NE3, KYSE30, TE13, KYSE510, EC109, KYSE140, EC9706, and AKR cells were sourced from Procell (Wuhan, China). HEK293 and AKR cells were cultured in Dulbecco's modified Eagle's medium, while NE3, KYSE30, TE13, KYSE510, EC109, KYSE140, and EC9706 cells were cultured in RPMI‐1640 medium, according to ATCC guidelines. For drug treatments, cells were exposed to 5 µM olaparib (Selleck) or 20 µM etoposide (Meilun Bio) for the indicated durations.

### 2.3 Vector construction and lentiviral infection

The DNA segments encoding HMGA1 and PARP1 were amplified from the genomic DNA of TE13 cells and subcloned into the lentiviral vector ENTi‐CRISPR v3‐HA‐Flag‐Puro (GenePharma). After 48 h, the cells were treated with 3 µg/mL puromycin (Cayman Chemical) to select successfully transfected clones. HMGA1/PARP1 expression in different clones was detected by qRT‐PCR. Additionally, the lentiviral vector GV654/G418 (GenePharma) was used to package short hairpin RNA (shRNA) for HMGA1/PARP1 knockdown. ShRNAs for HMGA1 were sourced by GenePharma, while shRNAs for PARP1 were sourced by Genechem. The sequences are listed in Table S2. After 48 h, the cells were treated with G418/puromycin and diluted for individual clone selection. The clones with the highest expression were used in subsequent experiments.

### 2.4 Modified small interfering RNA for transient knockdown

KYSE30, KYSE510, and TYK‐nu cells were transfected with custom‐made modified control and siRNA. The siRNA sequences for PARG, BRCA1, and BRCA2 are provided in Table S2. Transfections were performed using 30 nM oligonucleotides and Lipofectamine 2000 (#11668019, Invitrogen).

### 2.5 RNA isolation and quantitative RT‐PCR (qRT‐PCR) assay

Total RNA was obtained from the cells using TRIzol reagent (Thermo Fisher), and cDNA was synthesized using a reverse transcriptase kit (R323‐01, Vazyme). qRT‐PCR was performed as previously mentioned.^43^ Primer sequences are listed in Table S2.

### 2.6 Cytotoxicity and cell viability assays

The cells were planted on 96‐well plates with a density of 2000 cells per well and treated with drugs for 24 h. Cell viability and half‐maximal inhibitory concentrations (IC50) were assessed using the cell counting kit‐8 (CCK‐8) assay (DOJINDO, CK04).

### 2.7 Colony formation assay

ESCC cells were inoculated in six‐well plates with a density of 500 cells per well. Cells were incubated at 37°C and 5% CO_2_ for 12−17 days, fixed with 4% paraformaldehyde for 20 min and dyed with crystal violet for 30 min. The colonies with ≥50 cells were counted by ImageJ. All the experiments were carried out three times.

### 2.8 Red and green fluorescence reporting system for NHEJ

This reporting system is based on the pCMV‐mCherry‐EGFP plasmid. The plasmid was double‐digested using EcoRI and XhoI, and the sgRNA target sequence containing the PAM sequence was integrated between mCherry and EGFP. Without editing, only red fluorescence is expressed. Green fluorescence is expressed only when successful cutting occurs, followed by frameshift repair via the NHEJ pathway, allowing a one‐third probability of successful green fluorescent protein expression. Figure 2K shows the mechanism of action of the NHEJ fluorescence reporter plasmid.

### 2.9 Immunoblotting

The cells are collected and cleaved in the RIPA buffer. After quantification, the protein samples were boiled and deformed, separated with 10−12% SDS‐PAGE glue and transferred to a PVDF membrane (Millipore). Closed with 5% bovine serum albumin (BSA) for 2 h and incubated overnight. On the second day, the primary antibody was discarded, TBST was cleaned three times, the second antibody was incubated for 1 h, and TBST was cleaned three times for detection.

### 2.10 Co‐IP and mass spectrometry

Whole‐cell extracts were prepared, and corresponding antibodies were incubated with the extracts. After 8 h, the protein A/G beads were added, and the mixture was shaken for 2 h. After five washes with precooled buffer, 20 µL of 1× loading buffer was added. SDS‐PAGE was used to identify interacting proteins or the samples were stained with Coomassie Brilliant Blue for mass spectrometry.^44^

### 2.11 Cellular PARylation assays

The cells were lysed in 4% SDS buffer and treated with ultrasound. The lysate was diluted with 1% NP‐40 lysate to prevent the PARylated protein from binding to the anti‐PAR antibody. The lysates were immunoprecipitated with an anti‐GFP antibody or anti‐PAR antibody. Western blot analysis of immunoprecipitated proteins was performed using anti‐PAR or anti‐GFP antibodies.^45^

### 2.12 Protein purification

The pET28a‐sumo vector carrying the HMGA1 fragment was transformed into TOP10F chemically competent cells. After induction of protein expression with IPTG, the cells were lysed, and the supernatant was isolated. The target protein was purified using a nickel column and washed with deionized water and binding buffer until no protein was detected.

### 2.13 Chromatin isolation

Chromatin fraction assays were performed as described previously^46^ with minor modifications. Cells were treated with 1 mM methyl methanesulfonate for 1 h, washed, and processed for chromatin isolation.

### 2.14 Chromatin immunoprecipitation assay

The cells were crosslinked with 1% formaldehyde followed by incubation with .125 M glycine to terminate crosslinking. Cells were collected, lysed, and subjected to ultrasound. An anti‐GFP antibody was used to immunoprecipitate the protein–DNA complex. After washing and elution, the enriched complex was analyzed by PCR and agarose gel electrophoresis.^47^

### 2.15 Immunofluorescence

Cells were fixed with 4% paraformaldehyde at 4°C for 30 min, blocked with 3% BSA, incubated with primary antibody for 4 h. After washing, incubated with corresponding secondary antibody for 2 h. After washing, DAPI staining the nucleus for 10 min. Images were obtained by laser scanning confocal microscopy (Zeiss, LSM980) and analyzed with the ImageJ software. A total of 100 cells were counted and the immunofluorescence expression rate was calculated.

### 2.16 Human ESCC tissue specimen

All the specimens were collected from tumours and adjacent normal tissues in Anyang People's Hospital, Beiguan District, Anyang City, Henan Province. All patients were diagnosed with ESCC. The use of these tissue samples was approved by the Ethics Committee of Henan University.

### 2.17 Hematoxylin and eosin (HE) staining

Tissues were dewaxed in xylene, followed by hydration in ethanol at various concentrations. After staining with hematoxylin and eosin, tissues were dehydrated using a series of ethanol concentrations. The slides were soaked in xylene and sealed with neutral resin.

### 2.18 Immunohistochemistry

After paraffin‐embedded tissue sections, xylene was dewaxed and graded ethanol was hydrated. The slices were soaked in .01 M sodium citrate or EDTA buffer and heated by microwave at high temperature for 6 min and medium‐high temperature for 6 min. After being restored to room temperature, they were blocked with goat serum and incubated at 4°C overnight with antibodies. The slices were then incubated with biotin and 3,3′ ‐diaminobenzidine tetrachloric acid (DAB) for 1 min. The nucleus was reversed and stained with hematoxylin.

### 2.19 Tissue Immunofluorescence

The tissue sections were fixed for 30 min and then washed with PBST five times for 5 min each time. Sections were closed with 3% BSA for 2 h and incubated with primary antibody at 4°C overnight. After washing, incubate with the corresponding secondary antibody in the dark for 2 h. Confocal microscopy was performed by DAPI staining.

### 2.20 Murine subcutaneous tumour models

Mouse esophageal carcinoma‐derived AKR (WT‐AKR) cells and HMGA1‐knocked down AKR (shHMGA1‐AKR) cells were injected subcutaneously into the axilla of mice. Tumour sizes were measured daily. Starting on day 3 post‐injection, mice were administered 50 mg/kg olaparib intraperitoneally every other day. After 2 weeks, the mice were sacrificed, and the tumours were excised, photographed, weighed, and measured. The tumour specimens were immobilized in formalin for 48 h and processed for histological analysis. All animal testing procedures are approved by the Institutional Animal Care and Use Committee (IACUC) of Henan University and are carried out in accordance with the Guidelines for the Care and Use of Experimental Animals of the Chinese Association of Experimental Animals.

### 2.21 Induction and treatment of ESCC in WT (Hmga1flox/flox) and HMGA1 conditional KO (Hmga1flox/floxK14) mice

Using CRISPR/Cas9‐mediated genetic engineering, whole‐body epithelial cell‐specific Hmga1 knockout mice were constructed in C57BL/6J mice. The phenotypes were identified, and all positive founder mice were provided by Cyagen Biotechnology. Six‐week‐old WT and HMGA1 KO mice (20–23 g) were treated with 4‐nitroquinoline N‐oxide (4NQO) to induce ESCC. Mice were randomly divided into groups and treated with olaparib (50 mg/kg, i.p.) every other day for 3 months, starting from the second month after 4NQO induction. Esophageal tissues were collected for further analysis. All animal testing procedures are approved by the IACUC of Henan University and are carried out in accordance with the Guidelines for the Care and Use of Experimental Animals of the Chinese Association of Experimental Animals.

### 2.22 Single‐cell RNA‐seq data analysis

ESCC single‐cell RNA sequencing data (GSE188900) were downloaded from the GEO database and re‐analyzed using the Seurat software package to compare HMGA1 and PARP1 expression levels in different ESCC cell types. The analytical method follows the previously reported scheme.^48^, ^49^

### 2.23 Statistical analysis

All data were analyzed using GraphPad Prism 8.0. and SPSS Statistics 22.0 Each experiment was repeated at least three times, and results are expressed as mean ± standard deviation (mean ± SD). Student's *t*‐test was used to analyze the differences between the two groups. Statistical significance was defined as follows: ns = no significant difference, *p < .05, **p < .01, ***p < .001, and ****p < .0001.

## 3 RESULTS

### 3.1 HMGA1 interacts with PARP1

**FIGURE 1:**
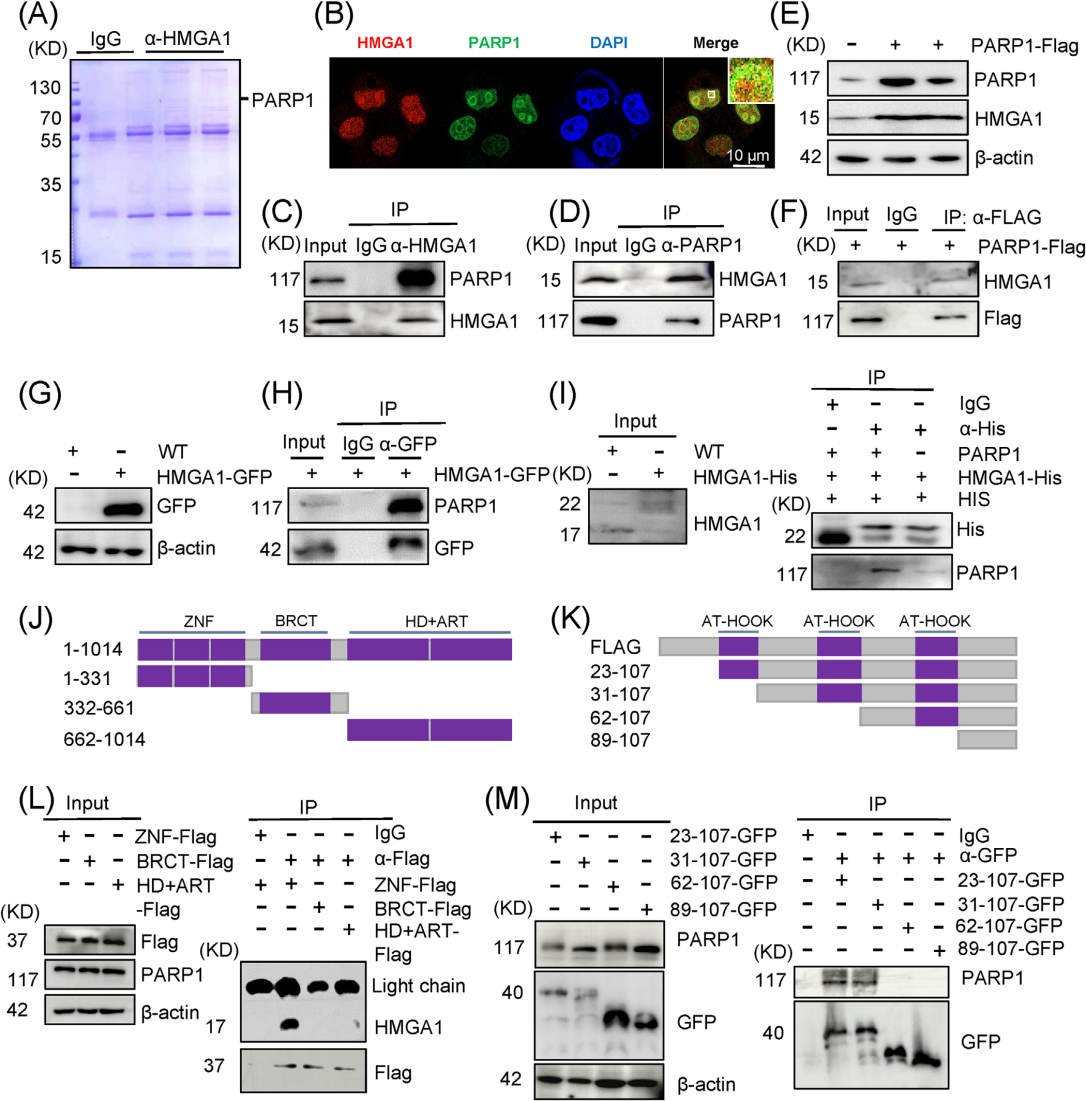
HMGA1 interacts with PARP1. (A) KYSE30 cell lysates were subjected to IP pull‐down experiments with anti‐HMGA1 antibody, followed by Coomassie blue staining after SDS‐PAGE, and analyzed by mass spectrometry (MS). (B) KYSE30 cells were treated with etoposide (ETO, 20 µM) for 2 h, stained with specific antibodies for immunofluorescence, and nuclei were labelled with DAPI. (C, D) KYSE30 cell lysates were subjected to IP pull‐down using anti‐HMGA1 or anti‐PARP1 antibodies, and the corresponding proteins were detected by Western blot (WB), *n* = 3. (E–H) IP pull‐down assays were performed using anti‐Flag and anti‐GFP antibodies, followed by WB detection for HMGA1 and PARP1, *n* = 3. (I) In vitro pull‐down assays were conducted using an anti‐His antibody after protein purification, *n* = 3. (J) PARP1 domain diagram: ART, ADP‐ribosyl transferase; BRCT, BRCA1 C‐terminal; HD, helical subdomain; ZF, zinc finger. (K) HMGA1 domain diagram showing the AT‐hook domain. (L) PARP1 domain deletions were incubated with KYSE‐30 whole cell lysate, and pull‐down proteins were analyzed by WB using an anti‐Flag antibody, *n* = 3. (M) HMGA1 domain deletions were incubated with KYSE‐30 whole‐cell lysate, and pull‐down proteins were analyzed by WB using an anti‐GFP antibody, *n* = 3.

HMGA1, a nonhistone chromatin structural protein, plays a regulatory role by modifying DNA structure. However, its additional functions in the nucleus remain largely uncharacterized. To explore the potential biological roles of HMGA1 in the nucleus and identify HMGA1‐mediated interactions, we extracted whole‐cell lysates from HMGA1‐overexpressing cells and performed co‐immunoprecipitation (co‐IP) using an anti‐HMGA1 antibody or control IgG. The immunoprecipitated proteins were subjected to liquid chromatography‐tandem mass spectrometry (LC‐MS/MS) (Figure 1A, Figure S1A, and Table S1). Among the identified proteins, besides the keratin family, XRCC5 (Ku80), XRCC6 (Ku70), and PARP1 were notable for their roles in DNA damage repair. Based on this, we hypothesized that HMGA1 might participate in DNA damage repair. Given that PARP1 is an early responder to DNA damage, we focused on the interaction between HMGA1 and PARP1.

Immunofluorescence staining revealed that HMGA1 and PARP1 co‐localized in the nucleus (Figure 1B). Reciprocal immunoprecipitation (IP) analysis in KYSE30 and KYSE510 cells using anti‐HMGA1 and anti‐PARP1 antibodies confirmed that HMGA1 co‐immunoprecipitated with PARP1, and vice versa (Figure 1C,D; Figure S1B). To further validate this interaction, we constructed KYSE30 cells expressing Flag‐tagged PARP1 and GFP‐tagged HMGA1. Co‐IP with anti‐Flag and anti‐GFP antibodies confirmed the reciprocal interaction between HMGA1 and PARP1 (Figure 1E–H). We also cloned HMGA1 into a prokaryotic expression system to obtain HMGA1 recombinant protein. An in vitro pull‐down assay demonstrated a direct interaction between HMGA1 and PARP1 (Figure 1I).

PARP1 consists of a C‐terminal catalytic domain containing spiral subdomains (HD) and ADP ribotransferase subdomains, a central self‐modifying domain containing BRCT motifs, and an N‐terminal DNA‐binding domain containing three ZNF motifs.^50^ To further characterize the interaction between HMGA1 and PARP1, we cloned Flag‐tagged PARP1 fragments into a eukaryotic expression system and performed co‐IP using a Flag antibody. We found that the region containing the PARP1 ZNF motif (amino acids 1−331) is essential for its interaction with HMGA1 (Figure 1J,L).

HMGA1 contains three AT‐hook domains rich in lysine and arginine residues. To identify which domain(s) of HMGA1 mediate its interaction with PARP1, we generated cell lines overexpressing GFP‐tagged AT‐hooks and other HMGA1 fragments and performed co‐IP using a GFP antibody. The study found that the region containing HMGA1 (amino acids 23−62) is required for the interaction with PARP1 (Figure 1K,M). These findings suggest that HMGA1 (23–62 amino acids) interacts with the PARP1 ZNF motif (1–331 amino acids).

### 3.2 HMGA1 responds to DNA damage and promotes DNA damage repair

**FIGURE 2:**
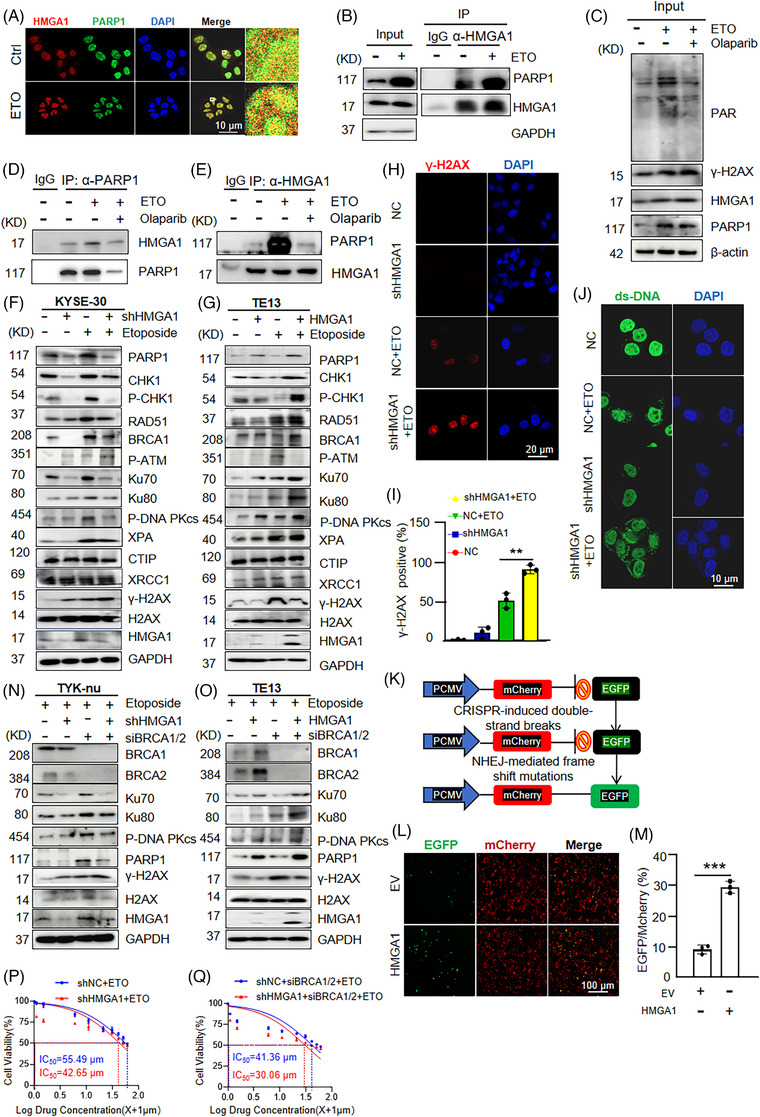
HMGA1 responds to DNA damage and promotes DNA damage repair. (A) Immunofluorescence shows the localization of HMGA1 and PARP1 following etoposide treatment. (B) Interaction between HMGA1 and PARP1 post‐etoposide treatment was detected by IP pull‐down using an anti‐HMGA1 antibody, *n* = 3. (C–E) After etoposide and olaparib treatment, the interaction between PARP1 and HMGA1 was detected by IP pull‐down using anti‐PARP1 and anti‐HMGA1 antibodies, respectively, compared with untreated cells, *n* = 3. (F) Stable HMGA1 knockdown KYSE‐30 cells were analyzed by WB to detect protein expression following etoposide treatment for 2h, *n* = 3. (G) HMGA1‐overexpressing stable TE13 cells were analyzed by WB to detect protein expression in response to etoposide (20 µM) treatment for 2 h, *n* = 3. (H, I) Control and etoposide‐treated (20 µM for 2 h) KYSE30 cells were stained with anti‐HMGA1 and γ‐H2AX antibodies, and nuclei were stained with DAPI. Immunofluorescence showed the accumulation of HMGA1 at DNA damage sites (H), with 100 cells counted to calculate the positive rate of HMGA1 localization (I). ***p < .001, *n* = 3. (J) KYSE30 cells were treated with etoposide for 2 h, followed by immunofluorescence staining using specific antibodies. Nuclei and double‐stranded DNA were labelled with DAPI and dsDNA markers. (K) Schematic representation of the pCMV‐mCherry‐EGFP NHEJ fluorescent reporter plasmid working principle. (L, M) HEK293 cells transfected with the pCMV‐mCherry‐EGFP NHEJ fluorescent reporter plasmid were used to detect the effect of HMGA1 mutation on NHEJ repair efficiency. Positive and negative cells in the visual field were counted, and the percentage of positive cells was calculated (M). *p < .05, **p < .01, ***p < .001, *n* = 3. (N) Control and HMGA1‐knockdown TYK‐nu cells were transfected with BRCA1/2 siRNA, and WB was performed to detect γ‐H2AX expression after etoposide (20 µM) treatment for 2 h, *n* = 3. (O) Control and HMGA1‐overexpressing TE13 cells were transfected with BRCA1/2 siRNA, and WB was performed to detect γ‐H2AX expression after etoposide (20 µM) treatment for 2 h, *n* = 3. (P, Q) Control and HMGA1 knockdown KYSE‐30 cells were transfected with BRCA1/2 siRNA and analyzed using the CCK8 assay, *n* = 3.

PARP1 is a key DNA damage response (DDR) protein involved in DNA repair.^32^, ^33^ To explore whether the interaction between HMGA1 and PARP1 influences DNA damage repair, we treated cells with the DNA‐damaging agent etoposide (ETO) and conducted immunofluorescence assays to examine the co‐localization of PARP1 and HMGA1. We observed increased accumulation of both PARP1 and HMGA1 in the nucleus of ETO‐treated cells (Figure 2A; Figure S2A). Consistently, co‐IP assays revealed that ETO treatment enhanced the interaction between PARP1 and HMGA1 (Figure 2B; Figure S2B,C).

As expected, ETO treatment increased the levels of poly(ADP‐ribose) (PAR) and γ‐H2AX, the latter being a surrogate marker for DNA damage (Figure 2C; Figure S2D). Pretreatment with olaparib reduced ETO‐induced PAR activation (Figure 2C; Figure S2D). Furthermore, olaparib treatment attenuated the ETO‐induced interaction between HMGA1 and PARP1 (Figure 2D,E; Figure S2E,F).

To investigate the role of HMGA1 in DNA damage, we treated ESCC cells with different concentrations of ETO and assessed the expression of HMGA1. ETO treatment led to a marked increase in γ‐H2AX and HMGA1 expression along with elevated levels of PARP1 and Ku70 at 20 µM ETO (Figure S2G). Time‐course experiments showed that HMGA1, PARP1, and Ku70 levels increased significantly at the early stages of DNA damage, peaking 180 min post‐ETO treatment (Figure S2H). Similarly, cisplatin (CDDP) treatment, which induces DNA damage, also increased the expression of HMGA1 in a dose‐ and time‐dependent pattern, with a peak at 25 µM CDDP after 10 h (Figure S2I,J). These findings suggest that HMGA1 responds to DNA damage and may play a role in DNA damage stress.

To further explore HMGA1's role in the DDR, we generated KYSE30 and KYSE510 cell lines with stable HMGA1 knockdown and treated them with ETO. In HMGA1‐knockdown cells, DNA damage markers γ‐H2AX and p‐ATM were significantly elevated, while HR pathway proteins (CHK1, p‐CHK1, BRCA1, BRCA2), NHEJ proteins (Ku70, Ku80, p‐DNA‐PKcs) were reduced. PARP1 and HMGA1 levels also decreased, while NER, BER, and Alt‐NHEJ pathway proteins (XPA, XRCC1, CTIP) remained unchanged (Figure 2F; Figure S2K). In contrast, HMGA1 overexpression in TE13 cells upregulated HR and NHEJ pathway proteins without affecting NER, BER, or Alt‐NHEJ proteins (Figure 2G; Figure S2K). Moreover, HMGA1 overexpression attenuated ETO‐induced DNA damage, as indicated by decreased γ‐H2AX and p‐ATM levels (Figure 2G; Figure S2K).

Immunofluorescence assays showed that HMGA1‐knockdown enhanced ETO‐induced γ‐H2AX foci and double‐stranded DNA accumulation in the cell (Figure 2H–J). Additionally, HMGA1 knockdown suppressed ETO‐induced signal enhancement of 53BP1, a reactive protein in the NHEJ pathway (Figure S2L,M).

Using an NHEJ fluorescence reporter plasmid (pCMV‐mCherry‐EGFP), we measured NHEJ repair efficiency. In cells co‐transfected with pCMV‐mCherry‐EGFP, sgRNA, and Cas, HMGA1 overexpression increased the number of EGFP‐positive cells, indicating enhanced NHEJ repair (Figure 2K–M). ETO treatment led to HMGA1 accumulation at DNA damage sites (Figure S2N,O).

To further confirm that HMGA1 promotes DNA repair via the NHEJ pathway, we knocked down BRCA1 and BRCA2 in TYK‐nu ovarian cancer cells to simulate a homologous recombination deficiency (HRD) state (Figure S2P). Under DNA damage conditions, HMGA1 knockdown increased DNA damage accumulation and inhibited NHEJ‐related protein expression (Figure 2N). Additionally, we assessed BRCA1 and BRCA2 expression in normal esophageal cells and EC cells and found that HMGA1, BRCA1, and BRCA2 were highly expressed in cancer cells (Figure S2Q). In BRCA1/BRCA2‐knockout TE13 cells, HMGA1 inhibited DNA damage accumulation and promoted NHEJ pathway protein expression (Figure 2O; Figure S2R).

To evaluate the effect of these conditions on cancer cell growth, we conducted CCK8 assays in TYK‐nu cells with stable BRCA1/BRCA2 knockdown. HMGA1 knockdown significantly reduced cell survival under DNA damage conditions (Figure 2P,Q). We also observed that HMGA1 protein levels correlated with PARP1 protein levels, although qRT‐PCR revealed that HMGA1 had no regulatory effect on PARP1 at the transcriptional level (Figure S2S). Collectively, these results suggest that HMGA1 mitigates DNA damage by promoting DNA repair.

### 3.3 PARP1 induces the PARylation of HMGA1

**FIGURE 3:**
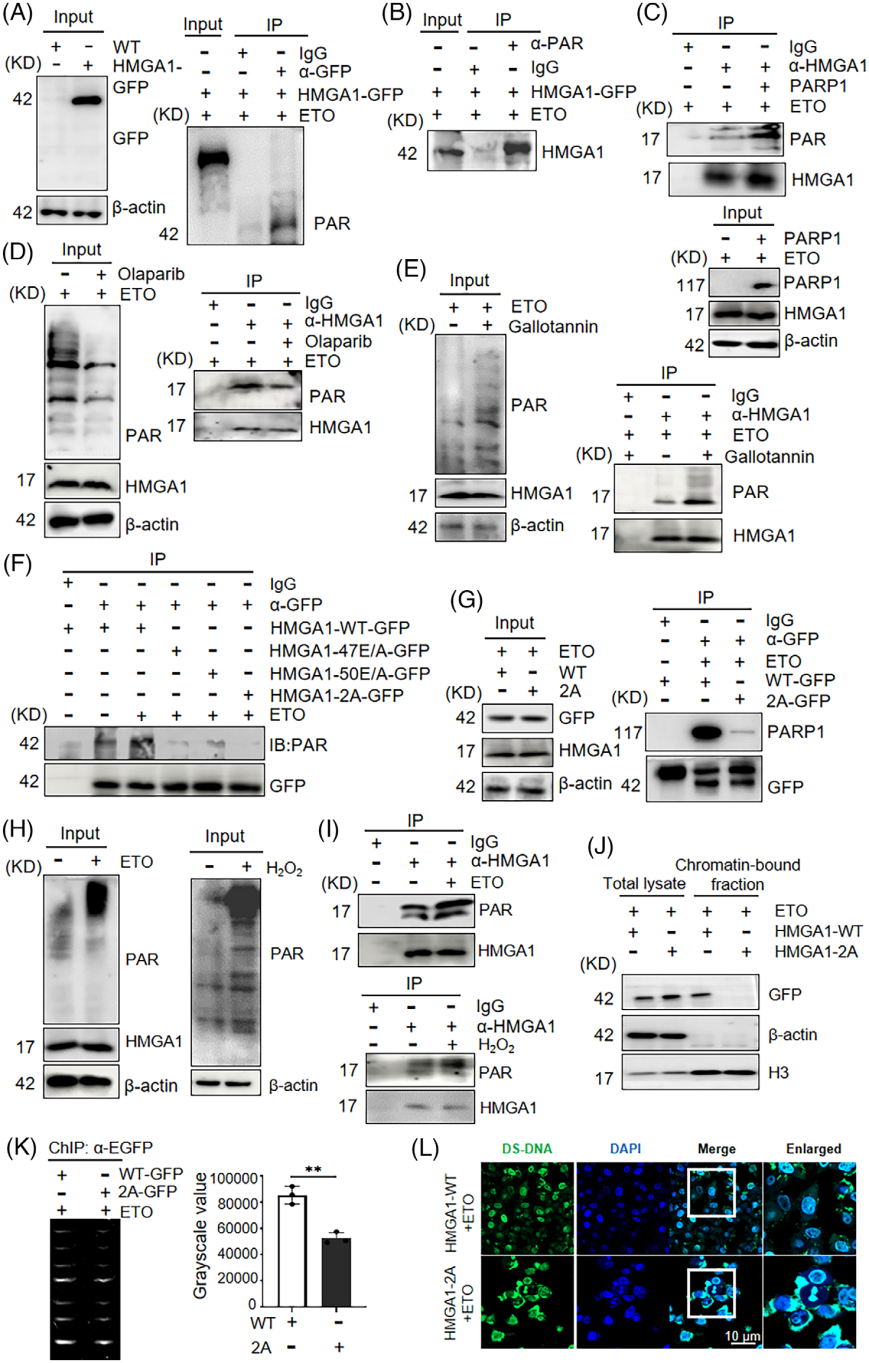
PARP1 induces the PARylation of HMGA1. (A, B) GFP‐HMGA1‐overexpressing cells were treated with etoposide (20 µM) for 2 h, lysed with 4% SDS buffer, and subjected to IP pull‐down using anti‐GFP (A) or anti‐PAR antibody, followed by WB detection with anti‐PAR/anti‐HMGA1 antibodies, *n* = 3. (C, D) IP pull‐down using anti‐HMGA1 antibodies detected PARP1 following etoposide (C) or olaparib (D) treatment, *n* = 3. (E) Cells were treated with gallotannin, followed by IP pull‐down using an anti‐HMGA1 antibody and WB detection for PARP1, *n* = 3. (F) PARylation of HMGA1 was detected in cells expressing PAR‐modified HMGA1 mutants, *n* = 3. (G) The effect of PAR modification on the interaction between HMGA1 and PARP1 was assessed using IP with an anti‐GFP antibody, *n* = 3. (H, I) HMGA1 PARylation was detected by co‐IP after etoposide (H) or H_2_O_2_ (I) treatment, *n* = 3. (J) Chromatin separation assay showed chromatin binding following the deletion of HMGA1 PAR modification, *n* = 3. (K) Chromatin binding of HMGA1 after deletion of PARylation was assessed using CHIP analysis, **p < .01, *n* = 3. (L) Double‐stranded DNA accumulation was detected in HMGA1 PAR‐modified cells using immunofluorescence.

PARP1 is involved in many cellular biological processes, including DNA damage repair, post‐translational modification of nuclear proteins through PARylation, and gene transcription.^22^ Next, we investigated whether PARylation also occurs in HMGA1. To determine if the PAR signal originates from HMGA1 or self‐modified PARP1, we used a 4% SDS buffer and 1% NP‐40 lysis buffer to eliminate the interaction between PARP1 and HMGA1 without affecting the covalent PARylation of HMGA1.^28^ As expected, HMGA1 (EGFP) pulled down the PAR signal, and PARylation of HMGA1 was observed, as indicated by a molecular weight smear starting at 17 kDa, confirming that the PAR signal came directly from HMGA1 (Figure 3A). Consistent with this finding, in cells with high basal PARP1 activity, we pulled down the PARylated protein using anti‐PAR antibodies and then performed immunoblotting using anti‐HMGA1 antibodies. The results confirmed that HMGA1 is indeed PARylated (Figure 3B). Furthermore, overexpression of PARP1 in cells increased HMGA1 PARylation (Figure 3C), while treatment with the PARP inhibitor olaparib reduced PARP1 activity and HMGA1 PARylation (Figure 3D). To confirm that PARP1 is responsible for HMGA1 PARylation, we used the specific PARP1 inhibitor AZD5305, which yielded similar results to olaparib, showing reduced PARylation of HMGA1 (Figure S3A). Additionally, treating cells with the PARG inhibitor gallotannin enhanced intracellular PARylation and increased HMGA1 PARylation (Figure 3E). Similarly, the application of PDD 00017273, another PARG‐specific inhibitor, also increased HMGA1 PARylation (Figure S3B).

Next, we aimed to identify the PARylation site(s) in HMGA1. PARylation typically occurs at glutamate (E), aspartic acid (D), tyrosine (Y), and serine (S) residues in conserved regions.^28^ Since HMGA1 interacts with PARP1 through amino acids 23−61 (Figure 1M), we compared conserved amino acids in this region across different species and found that E47 and E50 were conserved (Figure S3D). We constructed GFP‐HMGA1‐E47A and GFP‐HMGA1‐E50A mutants and overexpressed them in cells. Pull‐down assays using an anti‐GFP antibody followed by immunoblotting with a PAR antibody revealed a significant reduction in PARylation in the HMGA1‐E47A mutant and complete elimination of PARylation in the HMGA1‐E47A/E50A double mutant (Figure 3F). Moreover, the interaction between HMGA1‐E47A/E50A and PARP1 was significantly reduced compared with the interaction between wild‐type HMGA1 and PARP1 (Figure 3G). These findings suggest that HMGA1 is covalently modified by PARP1 via PARylation at the E47 and E50 sites.

To investigate the effect of DNA damage on HMGA1 PARylation, we treated cells with the DNA‐damaging agents etoposide (ETO) and hydrogen peroxide (H₂O₂), both known to activate PARP1.^25^ Co‐IP analyses showed that both ETO (Figure 3H) and H₂O₂ (Figure 3I; Figure S3C) enhanced HMGA1 PARylation. Given that HMGA1 functions as a dynamic regulator of chromatin remodelling, we explored the effect of HMGA1 PARylation on this function. Chromatin fractionation assays demonstrated that ETO treatment led to the accumulation of HMGA1 in the chromatin‐bound fraction, whereas the PARylation‐defective HMGA1‐2A mutant did not accumulate in the chromatin (Figure 3J). Chromatin immunoprecipitation assays further showed that the amount of DNA bound to HMGA1 decreased in the PARylation‐defective HMGA1‐2A mutant under ETO treatment (Figure 3K). Additionally, immunofluorescence assays revealed a significant increase in cytoplasmic double‐stranded DNA in cells expressing the PARylation‐defective mutant HMGA1‐2A (Flag/HMGA1‐E47A/E50A) compared with those expressing wild‐type HMGA1 (Figure 3L; Figure S3E). These results suggest that the accumulation of HMGA1 at DNA damage sites is dependent on PARylation. PARP1 mediates the PARylation of HMGA1 following DNA damage.

### 3.4 PARylation of HMGA1 promotes DNA damage repair by recruiting Ku70/Ku80

**FIGURE 4:**
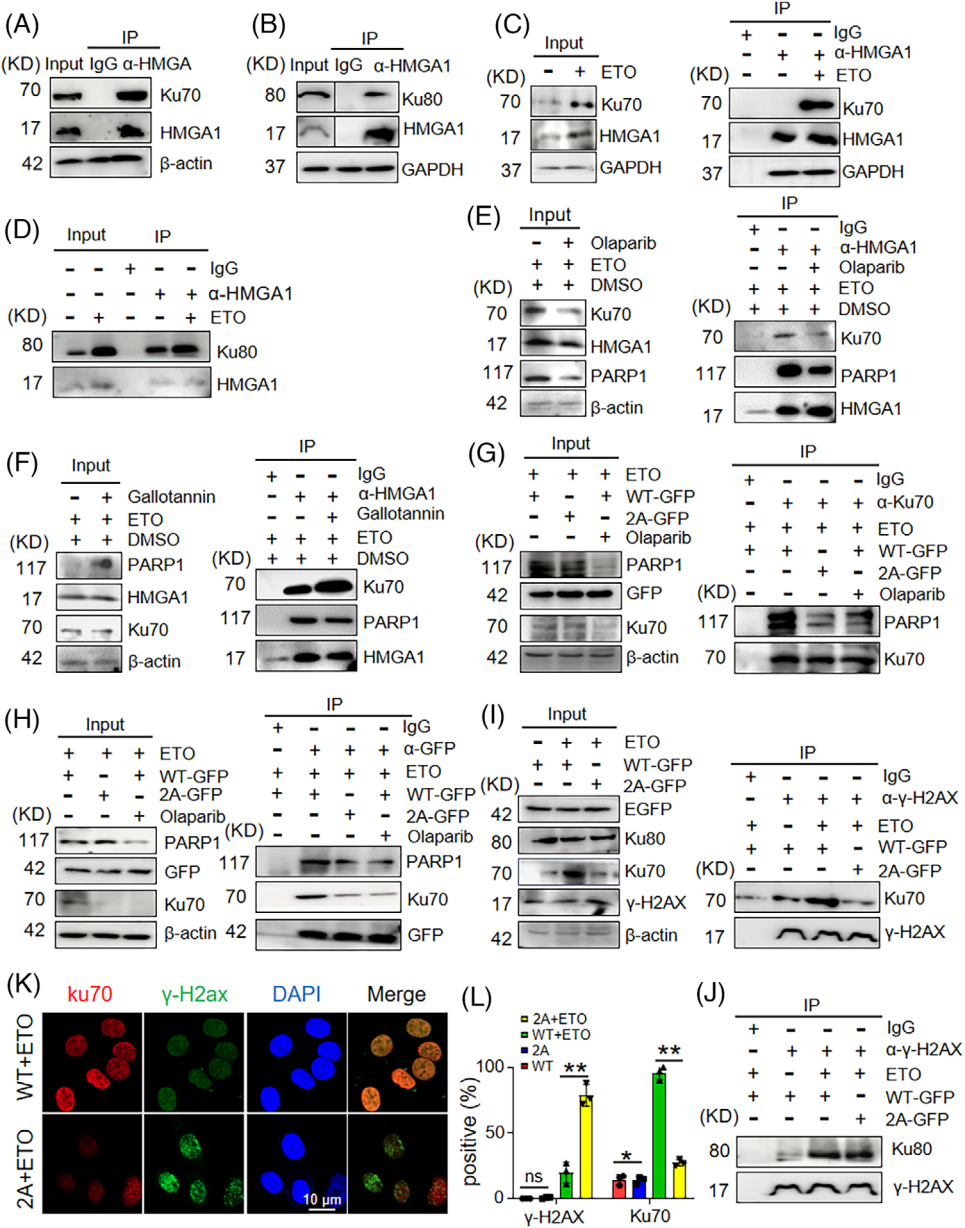
PARylation of HMGA1 promotes DNA damage repair by recruiting Ku70/Ku80. (A, B) IP pull‐down using an anti‐HMGA1 antibody detected the interaction between HMGA1 and Ku70 (A) or Ku80 (B), *n* = 3. (C, D) Following etoposide or PBS treatment, IP pull‐down using an anti‐HMGA1 antibody detected the interaction between HMGA1 and Ku70/Ku80, *n* = 3. (E, F) The interaction between HMGA1 and Ku70 following olaparib (E) or gallotannin (F) treatment was detected using IP pull‐down assays, *n* = 3. (G) WT‐HMGA1 or 2A‐HMGA1‐overexpressing cells were treated with etoposide and olaparib, and the interaction between Ku70 and PARP1 was assessed using IP pull‐down and WB, *n* = 3. (H) IP pull‐down using an anti‐GFP antibody detected the interaction between HMGA1‐GFP, Ku70, and PARP1 following etoposide and olaparib treatment in WT‐HMGA1 or 2A‐HMGA1‐overexpressing cells, *n* = 3. (I, J) IP pull‐down using an anti‐γ‐H2AX antibody detected the interaction of γ‐H2AX with Ku70 and Ku80 in WT‐HMGA1 or 2A‐HMGA1‐overexpressing cells, *n* = 3. (K, L) Immunofluorescence analysis of Ku70 and γ‐H2AX expression in WT‐HMGA1 or 2A‐HMGA1‐overexpressing cells after etoposide treatment (K) and 100 cells were counted and calculated (L). *p < .05, **p < .01, *n* = 3.

To investigate how HMGA1 promotes DNA damage repair, we examined its role in recruiting DDR repair factors. LC‐MS/MS analysis revealed that HMGA1 interacts with Ku70/Ku80 (Figure S1A). To validate this interaction, we transduced HA‐HMGA1 into cells and exposed them to ETO. Co‐IP assays confirmed the interaction between HMGA1 and Ku70/Ku80, and this interaction was substantially enhanced in ETO‐treated cells (Figure 4A,B; Figure S4A). Immunofluorescence assays showed that Ku70 accumulated at DNA damage sites, co‐localizing with γ‐H2AX, with increased accumulation and co‐localization in ETO‐treated cells (Figure S4B,C).

To determine whether PARylation influences the interaction between HMGA1 and Ku70/Ku80, we treated cells with the PARP1 inhibitor olaparib to suppress PARylation, or the PARG inhibitor gallotannin to enhance it. Olaparib treatment reduced the interaction between HMGA1 and Ku70 (Figure 4E), while gallotannin treatment increased it (Figure 4F). Using the PARP1‐specific inhibitor AZD5305 and the PARG‐specific inhibitor PDD 00017273 yielded similar results as treated with olaparib and gallotannin, confirming that PARylation affects the HMGA1‐Ku70 interaction (Figure S4D,E). Additionally, the lack of PARylation in HMGA1‐2A reduced the interaction between Ku70 and PARP1 compared with wild‐type HMGA1 (Figure 4G,F). Loss of HMGA1 PARylation also inhibited the formation of the HMGA1‐Ku70 complex (Figure 4H).

To assess whether HMGA1 PARylation affects Ku70/Ku80 recruitment to DNA damage sites, we transfected cells with wild‐type or PARylation‐deficient HMGA1 constructs and treated them with ETO. Immunoprecipitation assays with Ku70/Ku80 antibodies showed that the interaction between Ku70/Ku80 and γ‐H2AX was markedly reduced in cells expressing PARylation‐deficient HMGA1 mutants (Figure 4I,J). Immunofluorescence assays also demonstrated reduced co‐localization of Ku70/Ku80 and γ‐H2AX in cells expressing HMGA1‐E47A/E50A (Figure 4K,L; Figure S4G). These results indicate that HMGA1 promotes the accumulation of Ku70 to the sites of DNA damage, and this function depends on HMGA1 PARylation.

### 3.5 HMGA1 promotes DNA damage repair by upregulating DNAPKcs activation

**FIGURE 5:**
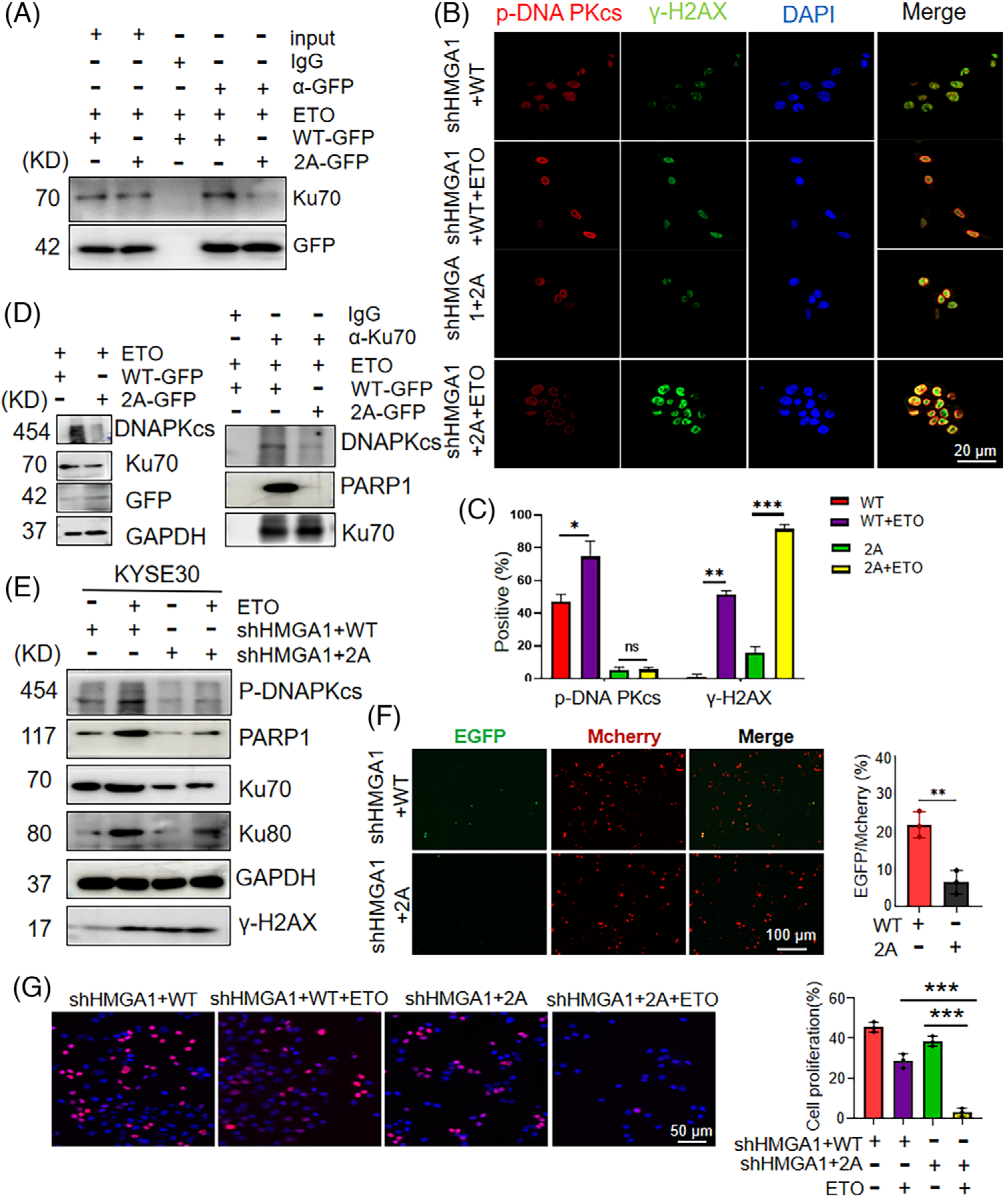
HMGA1 promotes DNA damage repair by upregulating DNAPKcs activation. (A) The interaction between HMGA1‐WT‐GFP, HMGA1‐2A‐GFP, and Ku70 with or without etoposide treatment was detected using an anti‐GFP antibody in a pull‐down test, *n* = 3. (B, C) The expression of γ‐H2AX and p‐DNA PKcs was detected by immunofluorescence in HMGA1‐WT (Flag/HMGA1) and HMGA1‐2A (Flag/HMGA1‐E47A/E50A) cells under etoposide treatment (B), and 100 cells were counted and calculated (C). *p < .05, **p < .01, ***p < .001, *n* = 3. (D) The interaction between DNAPKcs and Ku70 was detected by a pull‐down test using an anti‐Ku70 antibody in HMGA1‐WT and HMGA1‐2A cells, with etoposide treatment, *n* = 3. (E) Cell lysates were collected from HMGA1‐WT and HMGA1‐2A cells after etoposide treatment or no treatment, and the related proteins were detected by WB, *n* = 3. (F) HMGA1‐WT (Flag/HMGA1) and HMGA1‐2A (Flag/HMGA1‐E47A/E50A) cells were co‐transfected with the pCMV‐mCherry‐EGFP NHEJ fluorescent reporter plasmid to assess the effect of HMGA1 mutation on NHEJ repair efficiency. Positive and negative cells were counted, and the percentage of positive cells was calculated. **p < .01, *n* = 3. (G) The proliferation ability of HMGA1‐WT and HMGA1‐2A cells was detected using an EDU assay under etoposide treatment, ***p < .001, *n* = 3.

In NHEJ DNA repair, PARP1 activation leads to the accumulation of Ku70/Ku80 at the sites of DNA damage, forming a complex that activates DNAPKcs, thereby promoting NHEJ DNA repair.^11^ Consistent with the findings from PARylation inhibitor olaparib treatment (Figure 4E), depletion of HMGA1 PARylation (HMGA1‐2A) impaired its interaction with Ku70 (Figure 5A). To determine whether PARylation of HMGA1 affected the formation of the DNAPK complex, we treated cells with ETO and performed immunofluorescence assays to examine the accumulation of DNAPKcs to the sites of DNA damage. The results showed that DNAPKcs were activated and p‐DNA PKcs levels increased in HMGA1‐WT‐transduced cells in response to ETO treatment (Figure 5B,C). Co‐localization of p‐DNA PKcs with γ‐H2AX was also enhanced (Figure 5B). However, cells transfected with the PARylation‐deficient mutant HMGA1 (Flag/HMGA1‐E47A/E50A) exhibited reduced interaction between γ‐H2AX and DNAPKcs, regardless of ETO treatment (Figure 5B). Similarly, the interaction between Ku70 and DNAPKcs was diminished (Figure 5D).

To further investigate the impact of HMGA1 PARylation on NHEJ repair, we measured p‐DNA PKcs levels in WT and HMGA1‐E47A/E50A cells, with or without ETO treatment, using immunoblotting. Both Ku70 and p‐DNA PKcs were reduced in HMGA1‐E47A/E50A cells, indicating a loss of response to DNA damage agents (Figure 5E). We also assessed NHEJ DNA repair efficiency using the pCMV‐mCherry‐EGFP NHEJ fluorescent reporter. The results showed that NHEJ‐mediated formation of EGFP was higher in HMGA1‐WT‐transduced cells compared with HMGA1‐E47A/E50A cells, suggesting that HMGA1 PARylation enhances NHEJ DNA repair (Figure 5F).In addition, EDU staining showed that the recombination of HMGA1‐E47A/E50A into HMGA1 interference cells enhanced the sensitivity of cells to ETO (Figure 5G; Figure s5a). Cloning and CCK8 experiments demonstrated that the loss of PARylation of intracellular HMGA1 led to decreased cell proliferation and viability (Figure S5B–D). These results support our hypothesis that HMGA1 promotes NHEJ repair through PAR modification and DNAPK activation. Depletion of HMGA1 PARylation decreases NHEJ DNA repair and sensitizes cells to DNA damage agents.

### 3.6 HMGA1 enhances the resistance of cancer cells to olaparib

**FIGURE 6:**
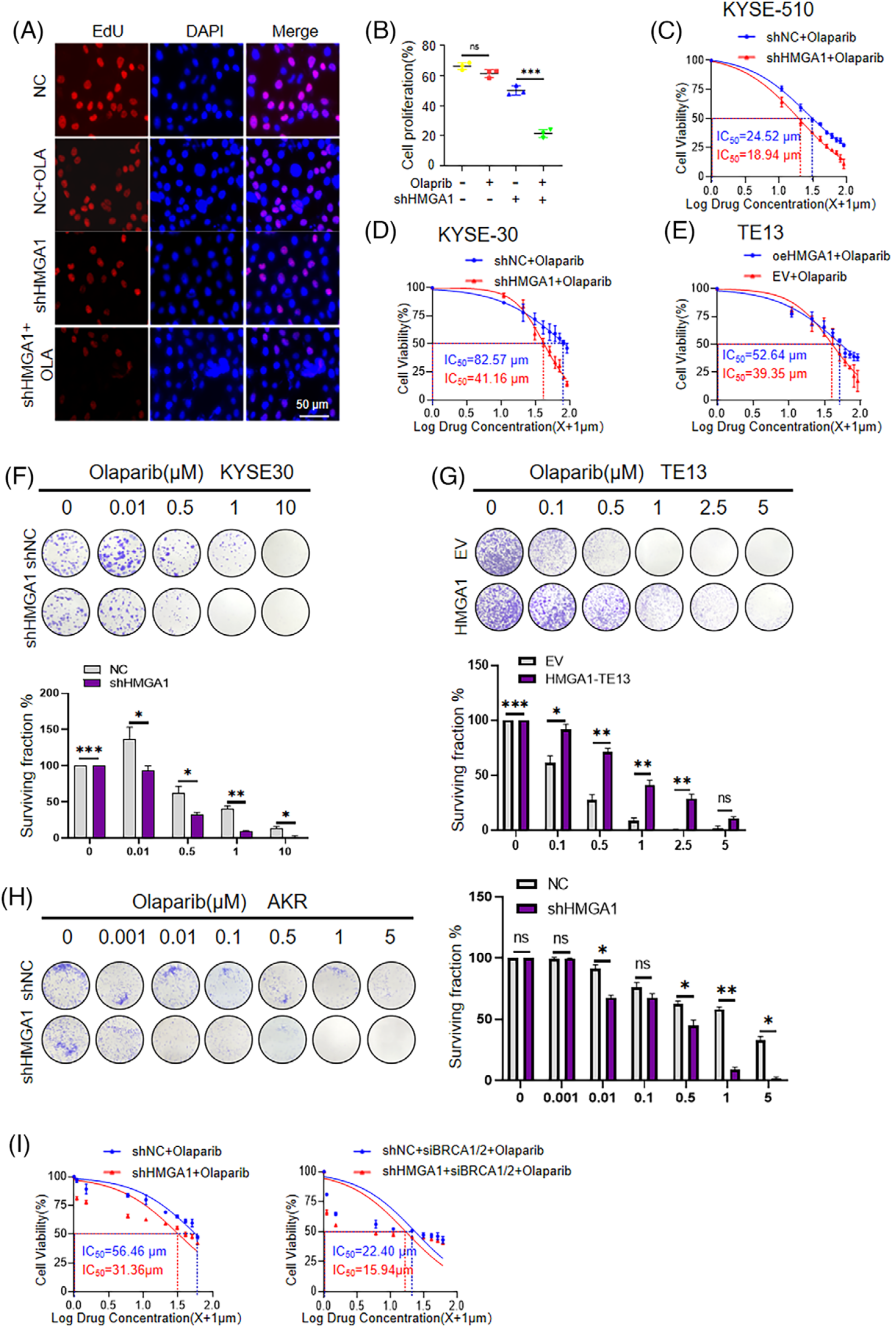
HMGA1 enhances cancer cell resistance to olaparib. (A, B) KYSE30 with or without HMGA1 knockdown were treated with or without olaparib, and cell proliferation was detected using an EDU assay (A). Data are presented with statistical analysis (B). ***p < .001, *n* = 3. (C–E) CCK8 assay was used to measure the survival of KYSE‐510, KYSE‐30, and TE13 cells with HMGA1 manipulations under olaparib stimulation, *n* = 3. (F–H) Colony formation assays were performed in KYSE‐30, KYSE‐510, and TE13 cells with HMGA1 manipulations. The colonies were calculated and shown, *p < .05, **p < .01, ***p < .001, *n* = 3. (I) CCK8 assay was used to measure cell survival under olaparib stimulation in HMGA1‐depleted TYK‐nu cells with BRCA1/2 siRNA, *n* = 3.

Since HMGA1 and PARP1 synergistically promote DNA damage repair, we hypothesized that inhibiting HMGA1 could sensitize tumour cells to PARP1 inhibitors like olaparib. To test this hypothesis, we first conducted an EDU incorporation assay in HMGA1‐manipulated cells with or without olaparib. HMGA1 knockdown enhanced olaparib's inhibitory effect on ESCC cell proliferation (Figure 6A,B). To further elucidate HMGA1's impact on ESCC susceptibility to olaparib, we used shRNA to knock down HMGA1 in KYSE510 cells, exposed the cells to olaparib, and measured the IC50 (the concentration at which 50% of cells cease proliferating) of the cells. Notably, HMGA1 depletion significantly increased the cells’ susceptibility to olaparib, with a marked decrease in the IC50 concentration compared with control KYSE510 cells (24.52 vs. 18.94 µM; *p* < .001; Figure 6C). Similarly, in KYSE30 cells, HMGA1 knockdown resulted in a significant reduction in the IC50 concentration (82.57 vs. 41.16 µM; *p* < .001; Figure 6D). Conversely, in TE13 cells with stable HMGA1 overexpression, we observed a significant increase in IC50 concentration (39.35 vs. 52.64 µM; *p* < .001; Figure 6E), indicating that HMGA1 overexpression promotes resistance to olaparib.

We then performed colony formation assays in KYSE30 cells with HMGA1 knockdown, AKR cells with HMGA1 knockdown, and TE13 cells with HMGA1 overexpression to further evaluate the sensitivity of HMGA1 to olaparib in ESCC cells. HMGA1 knockdown increased the cells’ susceptibility to olaparib, while overexpression increased resistance. To be specific, at the same olaparib concentration, shHMGA1 cells showed greater susceptibility to colony formation compared with control cells (Figure 6F,H). Conversely, HMGA1 overexpression in TE13 cells heightened resistance, as demonstrated by enhanced colony formation and cell viability compared with empty vector‐transduced cells under identical olaparib concentrations (Figure 6G).

Given that olaparib is clinically used to treat cancers with HRD, including ovarian and EC, we examined the sensitivity of HMGA1 to olaparib in HRD ovarian cancer cells. We knocked down BRCA1 and BRCA2 in ovarian cancer cells and treated them with olaparib in the presence or absence of HMGA1‐knockdown. CCK8 assays revealed that HMGA1 deletion enhanced the sensitivity of BRCA1/2‐depleted cells to olaparib with a lower IC50 concentration compared with HMGA1‐intact cells (22.40 vs. 15.94 µM; *p* < .001; Figure 6I).

### 3.7 Depletion of HMGA1 inhibits ESCC tumorigenesis and sensitizes tumours to olaparib

**FIGURE 7:**
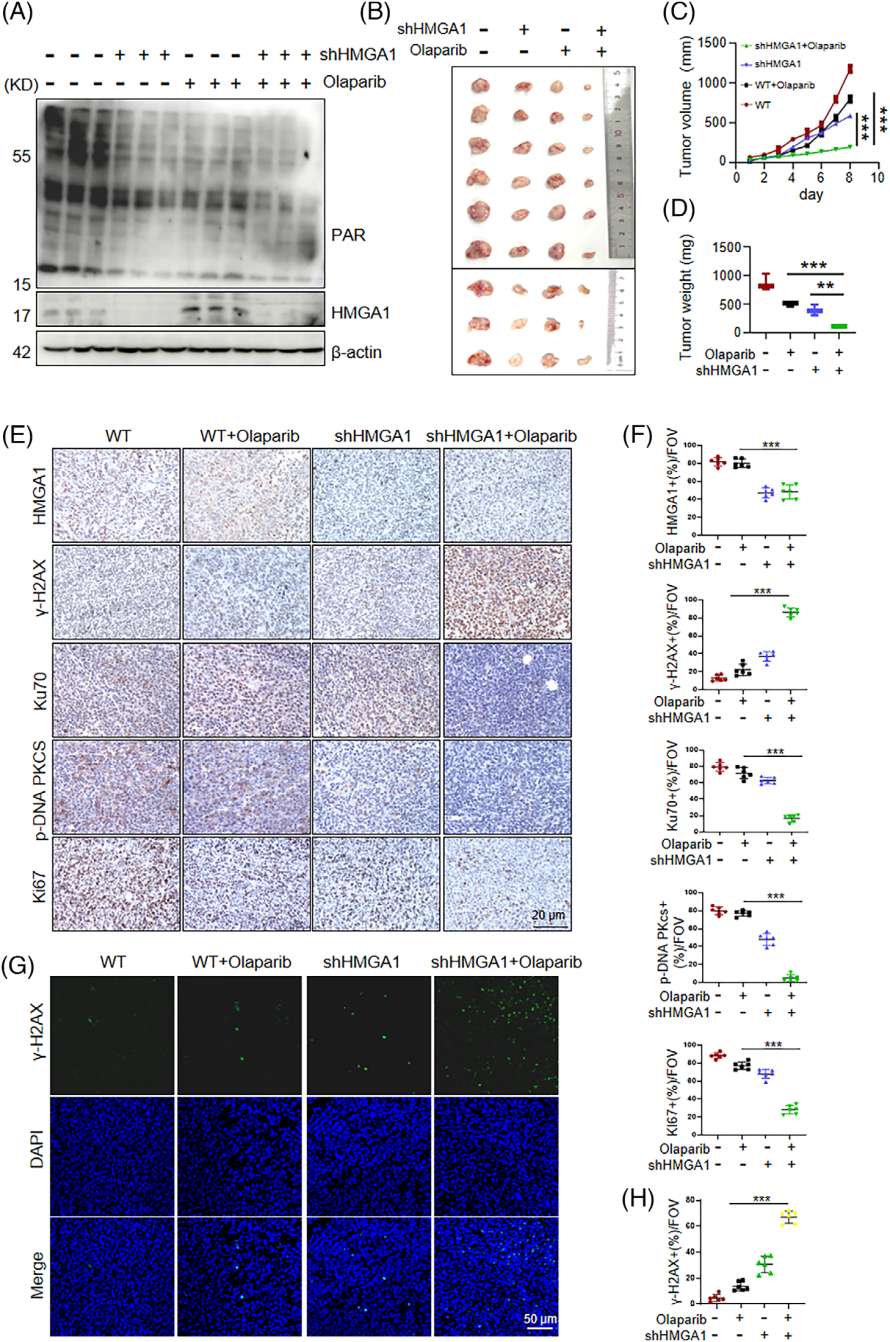
Depletion of HMGA1 inhibits ESCC tumorigenesis and sensitizes the tumours to olaparib. (A) After the dissection of the treated subcutaneous tumour, the tissue lysate was analyzed by WB, *n* = 3. (B–D) Statistical analysis of the size, volume, and weight of subcutaneous tumour tissues, **p < .01, ***p < .001, *n* = 6. (E, F) The expression and histological scores of HMGA1, γ‐H2AX, Ku70, Ki67, and p‐DNA PKcs were detected by IHC, ***p < .001, *n* = 6. (G, H) Immunofluorescence detection of γ‐H2AX expression in tissue samples, ***p < .001, *n* = 6.

To further assess the effect of HMGA1 depletion on PARP1 inhibition, we tested the effect of olaparib on tumours generated from HMGA1‐manipulated AKR cells in a syngeneic mouse model. Mice were subcutaneously inoculated with WT‐AKR cells or HMGA1‐knockdown AKR cells (shHMGA1‐AKR) and treated with olaparib (Figure 7A). Tumours from WT‐AKR cells were not significantly inhibited by olaparib (Figure 7B–D), whereas tumours with HMGA1 depletion showed marked inhibition with olaparib treatment (Figure 7B–D). While HMGA1 knockdown or olaparib alone failed to control tumour proliferation, their combination effectively inhibited tumour growth (Figure 7C,D). Immunohistochemistry revealed only weak upregulation of DNA damage markers (γ‐H2AX) due to HMGA1 interference alone, but olaparib treatment led to significant DNA damage in tumours with HMGA1 knockdown (Figure 7E,F). NHEJ repair proteins, Ku70 and p‐DNA‐PKcs, were also significantly decreased in tumours treated with both HMGA1 knockdown and olaparib.

Additionally, p‐ATM expression was increased in tumours with HMGA1 knockdown, and the combination of HMGA1 depletion and olaparib treatment further increased p‐ATM levels, suggesting excessive accumulation of DSB damage (Figure S6A). Tissue immunofluorescence confirmed that olaparib enhanced DNA damage in tumours with HMGA1 knockdown (Figure 7G). We also conducted similar experiments using the PARP1‐specific inhibitor AZD5305 and found that while AZD5305 alone had no significant effect, combining it with HMGA1 knockdown significantly inhibited tumour size, volume, and weight (Figure S6B–D).

### 3.8 HMGA1 knockout enhances the anti‐cancer effect of PARP1 inhibitors in an orthotopic ESCC model

**FIGURE 8:**
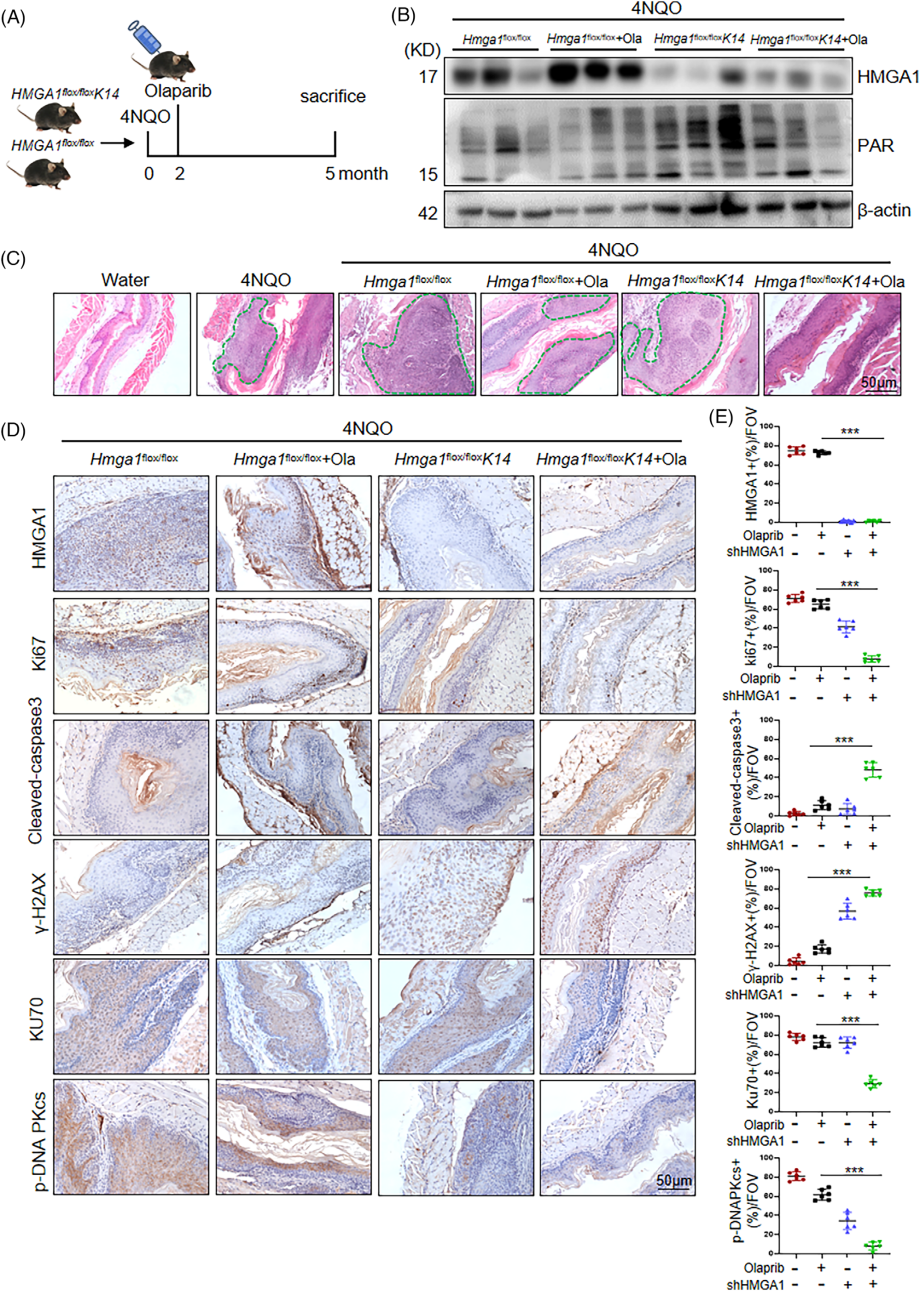
Depletion of HMGA1 inhibits ESCC tumorigenesis and sensitizes the tumours to olaparib. (A) Orthotopic ESCC was induced by 4NQO in WT (*Hmga1* ^flox/flox^) and esophageal‐specific HMGA1 knockout (*Hmga1* ^flox/flox^K14) mice, followed by olaparib therapy. (B) HMGA1 and PARylation protein levels in esophageal tissues were detected by WB, *n* = 3. (C) H&E staining was performed to detect tumour progression in esophageal tissues. (D, E) The expression and histological scores of HMGA1, Ki67, cleaved‐caspase 3, γ‐H2AX, Ku70, and p‐DNA PKcs were detected by IHC, ***p < .001, *n* = 6.

We next established a 4NQO‐induced orthotopic ESCC model using genetically engineered WT (Hmga1^flox/flox^) and esophageal‐specific HMGA1 knockout (Hmga1^flox/floxK14^) mice. Mice were treated with olaparib starting from the second month after 4NQO induction, and esophageal tissues were collected three months later (Figure 8A,B). H&E staining showed that 4NQO treatment induced ESCCs in Hmga1flox/flox (WT) mice, which were unresponsive to olaparib (Figure 8C). In contrast, ESCC formation was significantly reduced in Hmga1^flox/floxK14^ (HMGA1 knockout) mice, and this reduction was further enhanced by olaparib treatment (Figure 8C). Immunohistochemistry showed significant inhibition of tumour proliferation in Hmga1^flox/floxK14^ mice treated with olaparib, with a corresponding increase in apoptosis as indicated by cleaved caspase‐3 staining (Figure 8D,E). Additionally, the expression of Ku70 and p‐DNA PKcs was suppressed, and DNA damage markers (γ‐H2AX and p‐ATM) accumulated in tumours from Hmga1^flox/floxK14^ mice (Figure 8D,E; Figure S6E). These results demonstrate that HMGA1 is critical for ESCC tumorigenesis, and HMGA1 knockout enhances the anticancer effects of PARP1 inhibitors.

### 3.9 High HMGA1 expression in human esophageal cancer correlates with PARP1, Ku70, and p‐DNA PKcs expression

**FIGURE 9:**
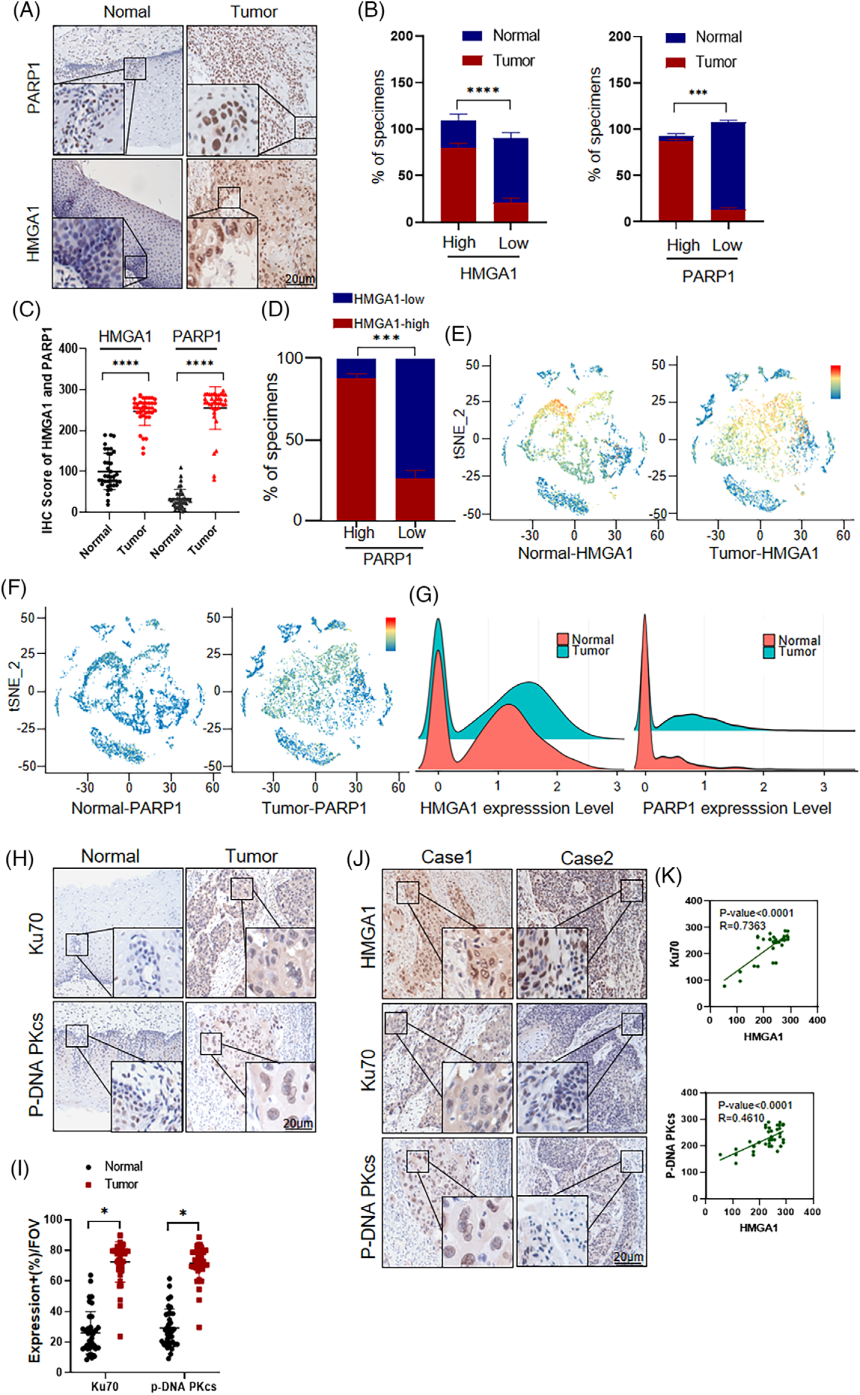
The high expression of HMGA1 in human ESCC is positively correlated with the expression of PARP1, Ku70, and p‐DNA PKcs. (A) The expression of HMGA1 and PARP1 was detected by IHC in cancer and para‐cancer tissue samples from 38 esophageal cancer patients. (B) The proportions of high and low HMGA1 and PARP1 expression were analyzed in normal and cancer tissue samples from 38 ESCC patients. (C) HMGA1 and PARP1 expression levels were analyzed in 38 ESCC patients. (D) The distribution of PARP1 expression in tissues with high and low HMGA1 expression was analyzed in 38 ESCC patients. (E, F) ESCC single‐cell RNA sequencing data (GSE188900) were downloaded from the GEO database and re‐analyzed using the Seurat software package to compare HMGA1 and PARP1 expression in different cell subtypes of ESCC tissues. (G) The same data were re‐analyzed to compare HMGA1 and PARP1 expression in normal and tumour cells. (H, I) Expression of Ku70 and p‐DNA PKcs in esophageal cancer and para‐cancerous tissues from 38 ESCC patients was detected by IHC, *p < .05, *n* = 38. (J, K) IHC and statistical analyses were performed to determine the correlation between the expression of Ku70, p‐DNA PKcs, and HMGA1 in ESCC patients, *n* = 38.

To explore the correlation between HMGA1 and PARP1 in ESCC patients, we analyzed HMGA1 and PARP1 expression in human esophageal tissues. Immunohistochemical (IHC) staining revealed low HMGA1 expression in normal esophageal tissues but a high expression in ESCCs, with a similar pattern observed for PARP1 (Figure 9A–C). A positive correlation was observed between HMGA1 and PARP1 expression (Figure 9D). Both HMGA1 and PARP1 were predominantly localized in the nucleus, consistent with their roles in DNA damage repair (Figure 9A).

We further analyzed single‐cell RNA sequencing data from ESCC and adjacent tissues in the GEO database (GSE188900). Using the Seurat package, we compared HMGA1 and PARP1 expression levels across different cell subtypes in ESCC tissues, such as immune cells, epithelial cells, and endothelial cells (Figure S7A). HMGA1 and PARP1 were primarily expressed in epithelial cells (Figure 9E,F). Further analysis using tSNE visualization confirmed that HMGA1 and PARP1 expression was significantly higher in cancer cells compared with adjacent noncancerous tissues (Figure 9G), consistent with the IHC results (Figure 9A).

We also evaluated the expression of Ku70 and p‐DNA PKcs in normal and ESCC tissues. Both proteins were expressed at low levels in normal esophageal tissues but were highly expressed in ESCCs (Figure 9H,I). When HMGA1 was abnormally elevated in ESCCs, Ku70 and p‐DNA PKcs expression was also increased (Figure 9J,K). These findings support the conclusion that HMGA1 and PARP1 are highly expressed in ESCCs, and that high HMGA1 expression is positively correlated with the expression of PARP1, Ku70, and p‐DNA PKcs.

## 4 DISCUSSION

EC is primarily composed of two distinct diseases, ESCC and EAC, with ESCC accounting for approximately 90% of cases.^1^ ESCC is the sixth leading cause of cancer‐related mortality worldwide, with more than half of cases occurring in China.^51^ It is an aggressive cancer that grows rapidly and has a high rate of metastasis.^52^ Symptoms such as cervical lymph node enlargement and dysphagia often manifest only when the cancer is advanced, resulting in poor prognosis and a low 5‐year survival rate.^53^

DNA damage‐based therapy is a cornerstone of cancer treatment. Unfortunately, tumour recurrence due to therapy resistance is common. HMGA proteins play a key role in cell transformation, and their overexpression is frequently observed in human malignancies, often correlating with poor prognosis and anti‐cancer resistance.^54^ Previous studies have demonstrated that the adaptor histone GH1‐HMGA1 in *Arabidopsis thaliana* is involved in telomere stability and DNA damage repair.^39^ HMGA1 activates the RAD51 promoter through two response elements, promoting radiation resistance, while its knockdown sensitizes clear cell adenocarcinoma cells to X‐ray exposure.^55^

Similarly, our study found that high HMGA1 expression in ESCC cells reduces DNA damage caused by ETO, while HMGA1 depletion increases DNA damage. Furthermore, we observed that HMGA1 upregulates the accumulation of both NHEJ and HR pathway repair proteins. Even after blocking the HR repair pathway, HMGA1 continued to promote DNA damage repair, reducing the accumulation of DNA damage. Post‐translational modifications (PTMs) of chromatin or chromatin‐associated proteins strictly regulate DDR and dynamically control protein stability, activity, and interactions.^56^ One critical PTM in DDR is PARylation, which is mainly caused by PARP1 catalysis. PARP1 accounts for approximately 90% of PAR production under genetic stresses.^57^ PARP1 is a key factor in DNA repair, participating in both c‐NHEJ and HR.^58^, ^59^ PARylation induces local chromatin recombination by modifying and recruiting chromatin‐modifying factors.^60^ While PARylation in DDR has been deeply studied, the biological functions of site‐specific PARylation remain poorly understood. Our study identified HMGA1 as a new PARylation partner for PARP1. We found that HMGA1 PARylation levels increased after genotoxic treatment, coinciding with PARP1 activation. Two residues in the conserved AT‐hook domain of HMGA1 (E47 and E50) were identified as PARylation sites. Mutations at these sites inhibited HMGA1's binding to PARP1.

HMGA1 is a chromatin‐remodelling factor that can alter chromatin structure. Interestingly, it has been reported that some chromatin‐related factors such as heterochromatin protein 1,^61^ SET domain bifurcation 1,^62^ and polycomb group proteins,^63^ are accumulated to the sites of DNA breaks and participate in DDR. These proteins undergo specific modifications that promote DNA repair by allowing local and transient chromatin relaxation.^64^ For example, SUV420H1‐mediated di‐ and tri‐methylation of histone H4 lysine 20 (H4K20me2/H4K20me3) plays an important role in DNA replication, repair, and heterochromatin formation.^65^ We found that HMGA1's chromatin‐binding ability was significantly reduced after mutation of its PAR modification sites.

Activation of PARP1 after DNA damage increases chromatin remodelling and initiates recruitment of DSB repair proteins, such as Ku70/Ku80 and XRCC4, via PAR or DNA binding.^66^ Ku70/Ku80 forms a dimer that recruits and activates DNAPK, promoting NHEJ repair. In our study, HMGA1 promoted the accumulation of Ku70/Ku80 and DNAPK to DNA damage sites through PAR modification, thereby enhancing NHEJ repair.

PARP has become a powerful target for cancer clinical treatment. PARPi work by causing genomic instability caused by replication, oxidative stress, and defects in DNA repair.^67^ PARPi significantly alters the therapeutic landscape of tumours with genetic defects, such as BRCA1 and BRCA2 mutations, which are primarily involved in the homologous repair of DNA damage.^68^ Olaparib, the first PARPi to gain regulatory approval in 2014, has been used as a maintenance therapy for recurrent ovarian cancer.^69^ Since 2009, multiple clinical trials have demonstrated the efficacy of PARPi against ovarian and breast cancers with BRCA mutations, as well as prostate, pancreatic, and small‐cell lung cancer.^70^ However, tumours that initially respond to PARPi often develop resistance through various mechanisms. Rational combination strategies are being explored to enhance PARPi efficacy and sensitize resistant tumours.

Olaparib resistance poses a major challenge in cancer treatment. In our experiments, subcutaneous tumour grafting and orthotopic EC models demonstrated that HMGA1 interference or gene knockout significantly inhibited EC tumorigenesis, an effect that was further enhanced by olaparib treatment. Abnormal accumulation of HMGA1 and PARP1 was observed in patient‐derived EC tissue, compared with relatively normal esophageal tissue, as well as in single‐cell data from ESCC and para‐cancerous tissues in the GEO database. These findings suggest that HMGA1 and PARP1 are associated with poor prognosis in ESCC patients.

However, some questions remain unresolved. It has been shown that overexpression of HMGA1 confers radioresistance by transactivating RAD51 in cholangiocarcinoma.^55^ In addition, the study showed that HMGA1 upregulates the expression of HR pathway protein, so whether HMGA1 also promotes HR repair needs further exploration. Additionally, targeting cell cycle checkpoint protein kinases, such as ATR, CHK1,^71^ and WEE1,^72^ has been shown to be a treatment for PARPi‐resistant cancers. Mechanically speaking, cell cycle checkpoint activation promotes cell cycle arrest, replication fork stabilization, and DNA repair, demonstrating the interaction of replication stress with DNA repair proteins in the development of PARPi resistance.^73^, ^74^, ^75^ In PARPi‐resistant ovarian and other cancers, inhibitors of these cell cycle checkpoints are being investigated.^76^ We found that EC with high HMGA1 expression is resistant to PARP inhibitors, and HMGA1 inhibition combined with PARP inhibitors is effective in treatment, or screening for ESCC patients with low HMGA1 expression may be effective against PARP inhibitors.

In conclusion, our study demonstrated that HMGA1 accumulates at the sites of DNA damage in a PARP1‐dependent pattern. We further characterized HMGA1 PARylation, which increased chromatin accessibility and promoted DNA repair by enhancing the recruitment of Ku70, Ku80, and DNAPK to damage sites. Finally, we showed that silencing HMGA1 enhanced tumour sensitivity to olaparib, providing a potential new approach to overcoming olaparib resistance in EC patients.

## AUTHOR CONTRIBUTIONS

Xin‐Yuan Lei designed the study and prepared the manuscript. Kai‐Yue He, Qiu‐Tong Li, Lei Zhang, Dan‐Hui Wu, Jing‐Yu Yang, Jin‐Rong Guo, Meng‐Jie Liu, Zi‐Long Zhao, Jun‐Qi Li, Huai Liu, Yuan Zhao, Yu‐jia Li, Qian‐Hui Sun, Chen‐Guang Wu, Yun‐Fan Wang, Geng‐Sheng Cao, and Gang Wang performed the experiments. Yong‐Ping Jian and Zhi‐Xiang Xu contributed to the study's conception and writing. All authors have read and approved the final manuscript.

## CONFLICT OF INTEREST STATEMENT

The authors declare no conflict of interest.

## CONSENT FOR PUBLICATION

All authors consent to the submission and publication of this article.

## Supporting information



Supporting information

Supporting information

Supporting information

Supporting information

Supporting information

Supporting information

Supporting information

Supporting Information

Supporting Information

## Data Availability

The authors declare that all data supporting the findings of this study are available in the article and its supplementary information files.
