# Supplementary figures and images for: PARylation of HMGA1 desensitizes esophageal squamous cell carcinoma to olaparib
Source: Clin Transl Med. 2024 Dec 17;14(12):e70111. doi: 10.1002/ctm2.70111 (PMC11652107; doi:10.1002/ctm2.70111)

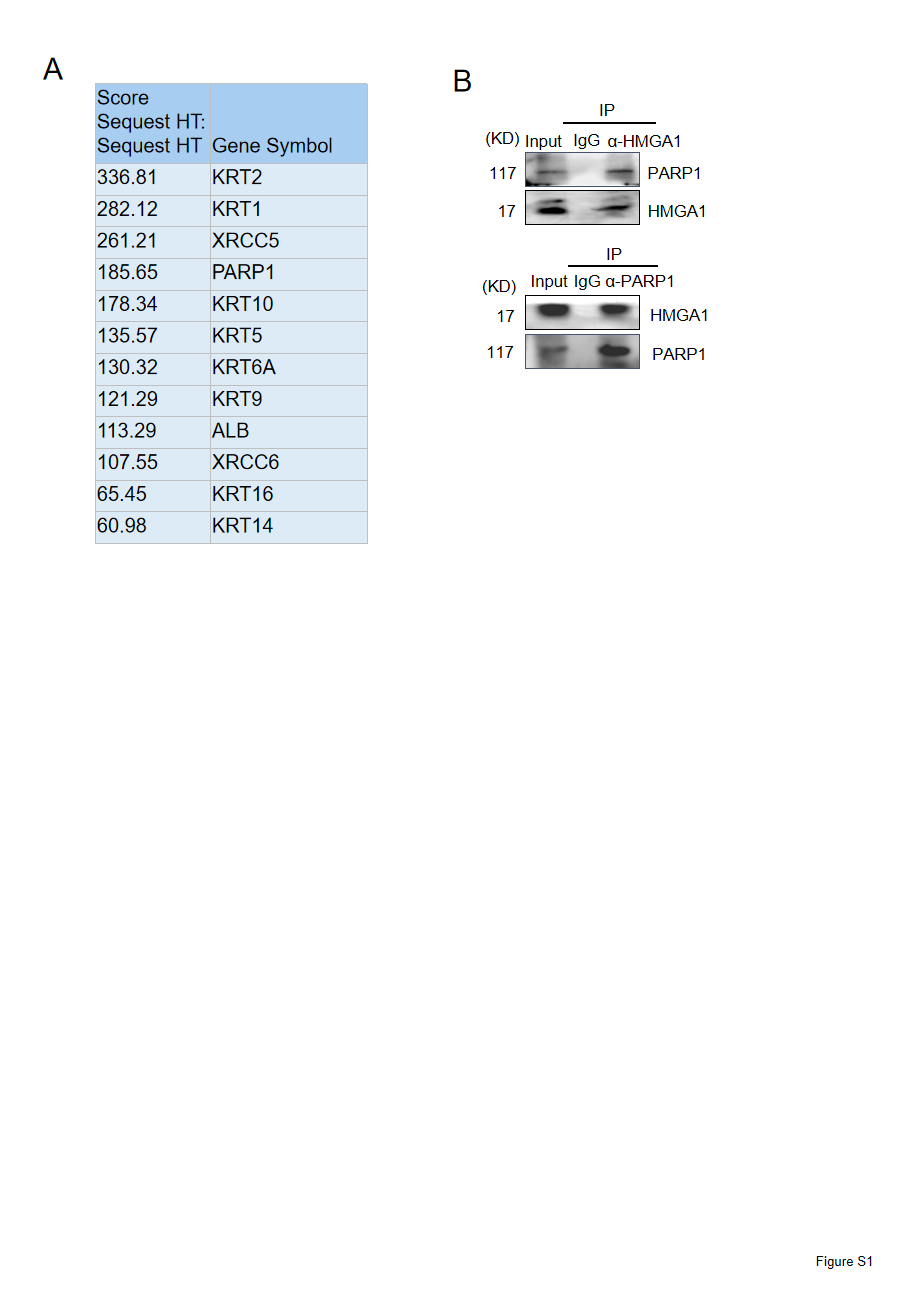

Supplement: Supplementary file 1 — Supporting information [file CTM2-14-e70111-s007.tif]

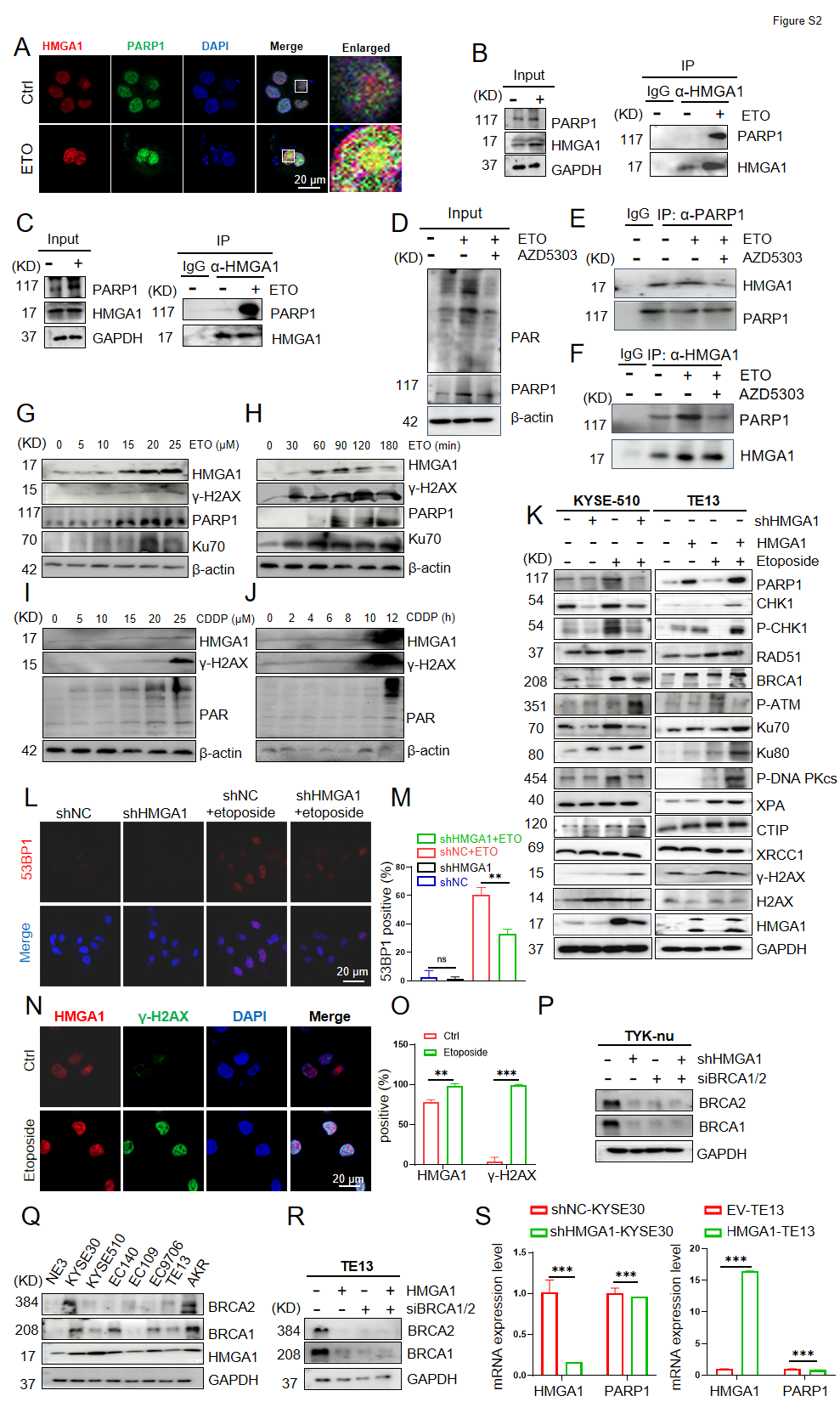

Supplement: Supplementary file 2 — Supporting information [file CTM2-14-e70111-s009.tif]

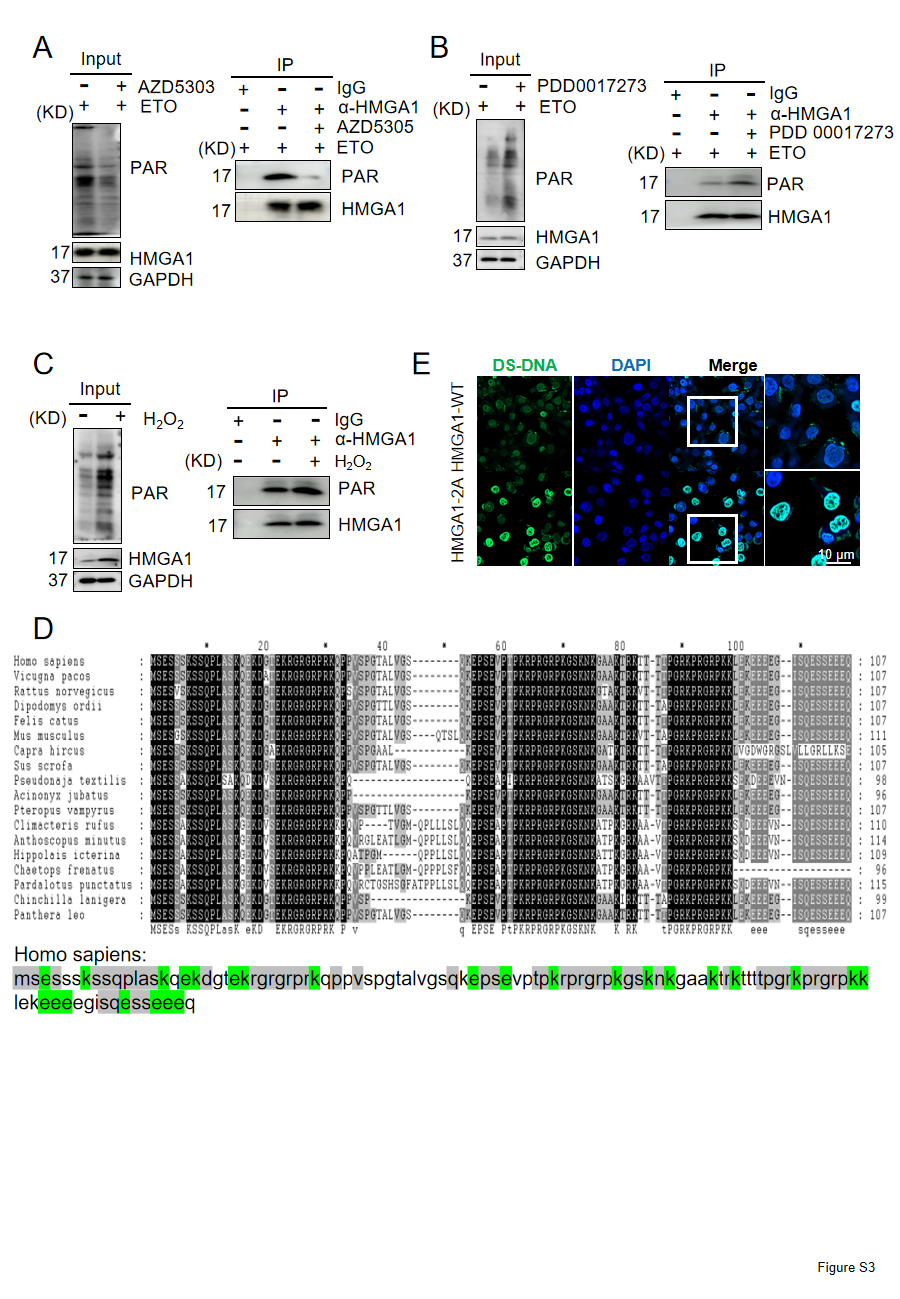

Supplement: Supplementary file 3 — Supporting information [file CTM2-14-e70111-s004.tif]

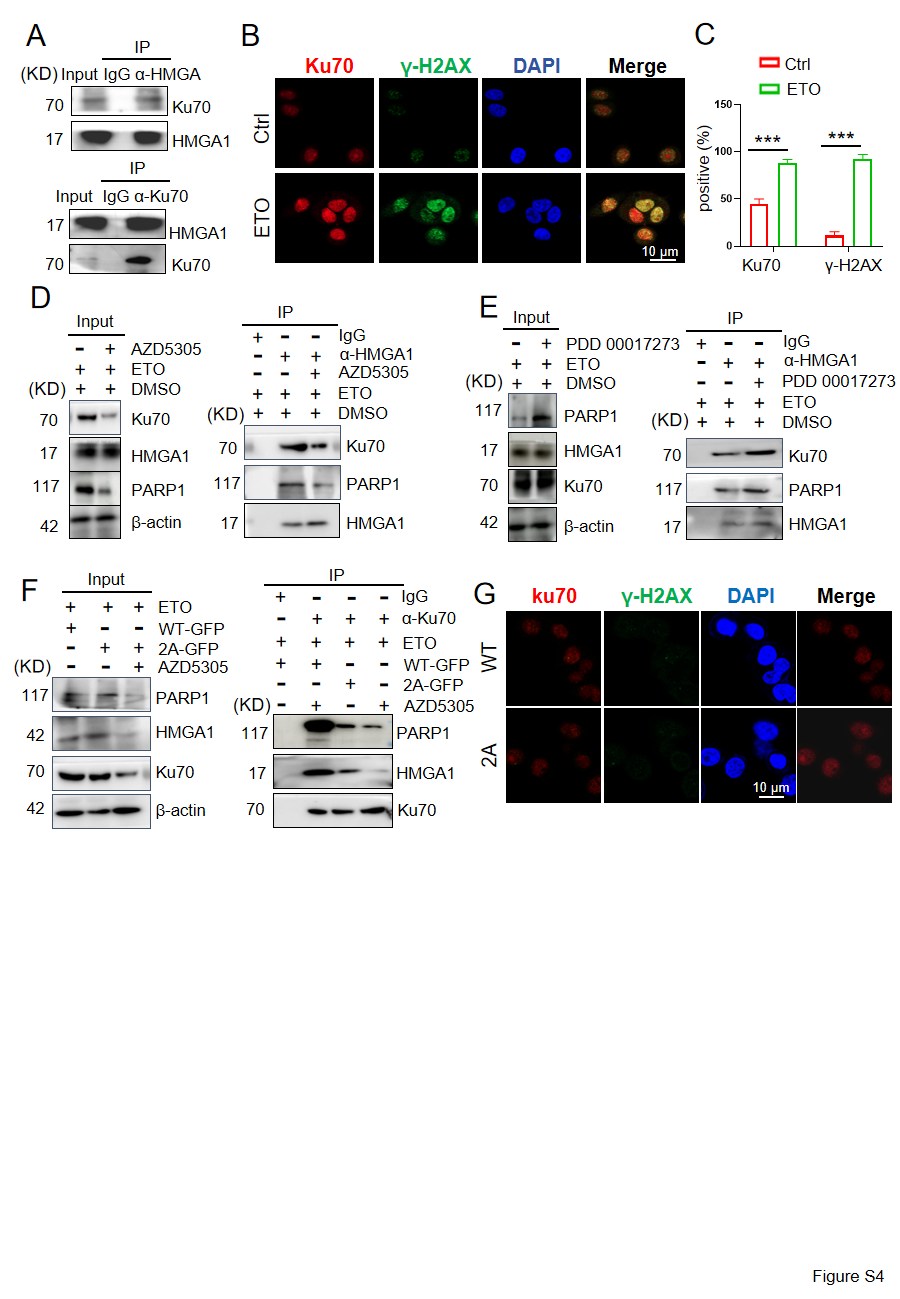

Supplement: Supplementary file 4 — Supporting information [file CTM2-14-e70111-s002.tif]

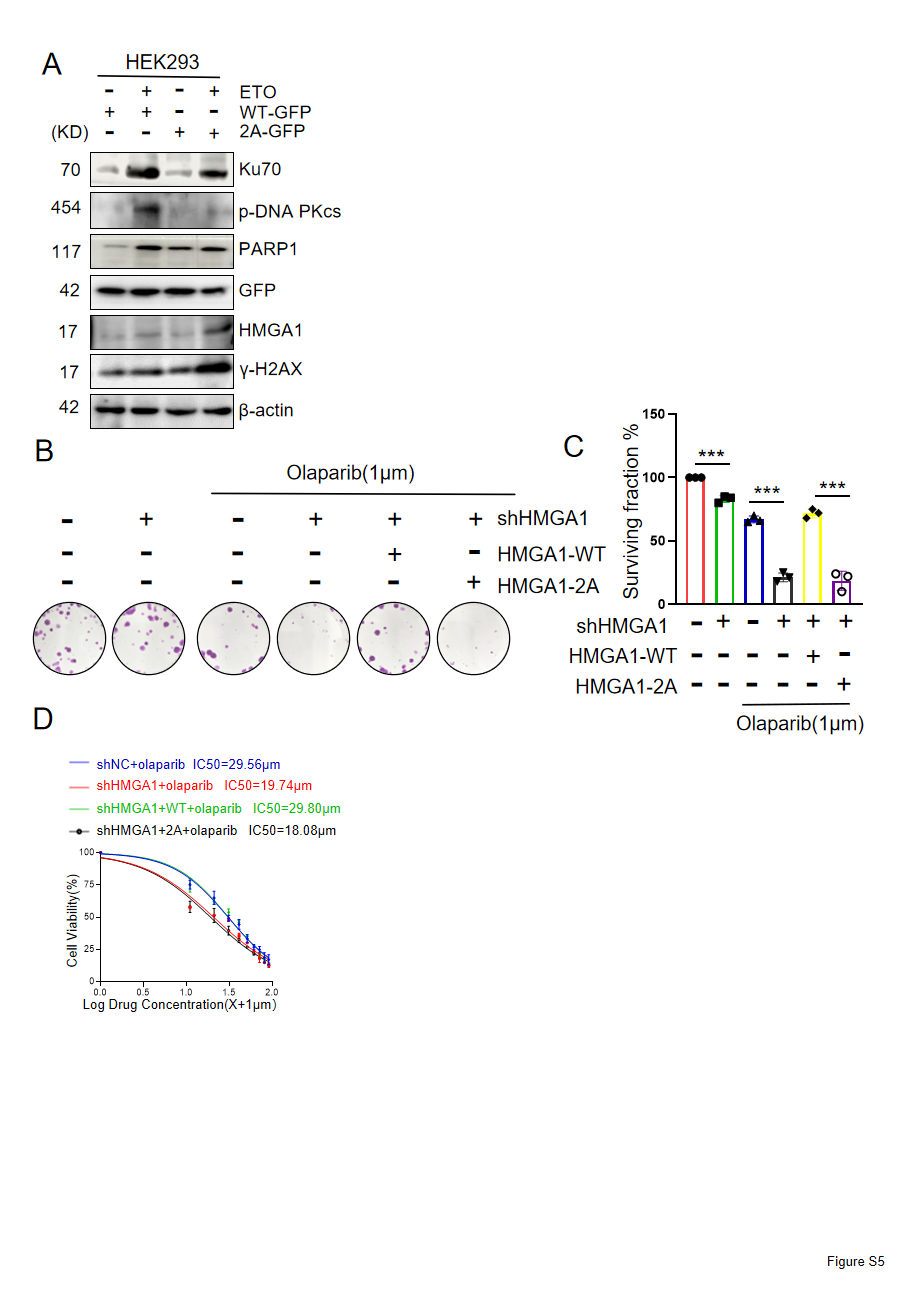

Supplement: Supplementary file 5 — Supporting information [file CTM2-14-e70111-s005.tif]

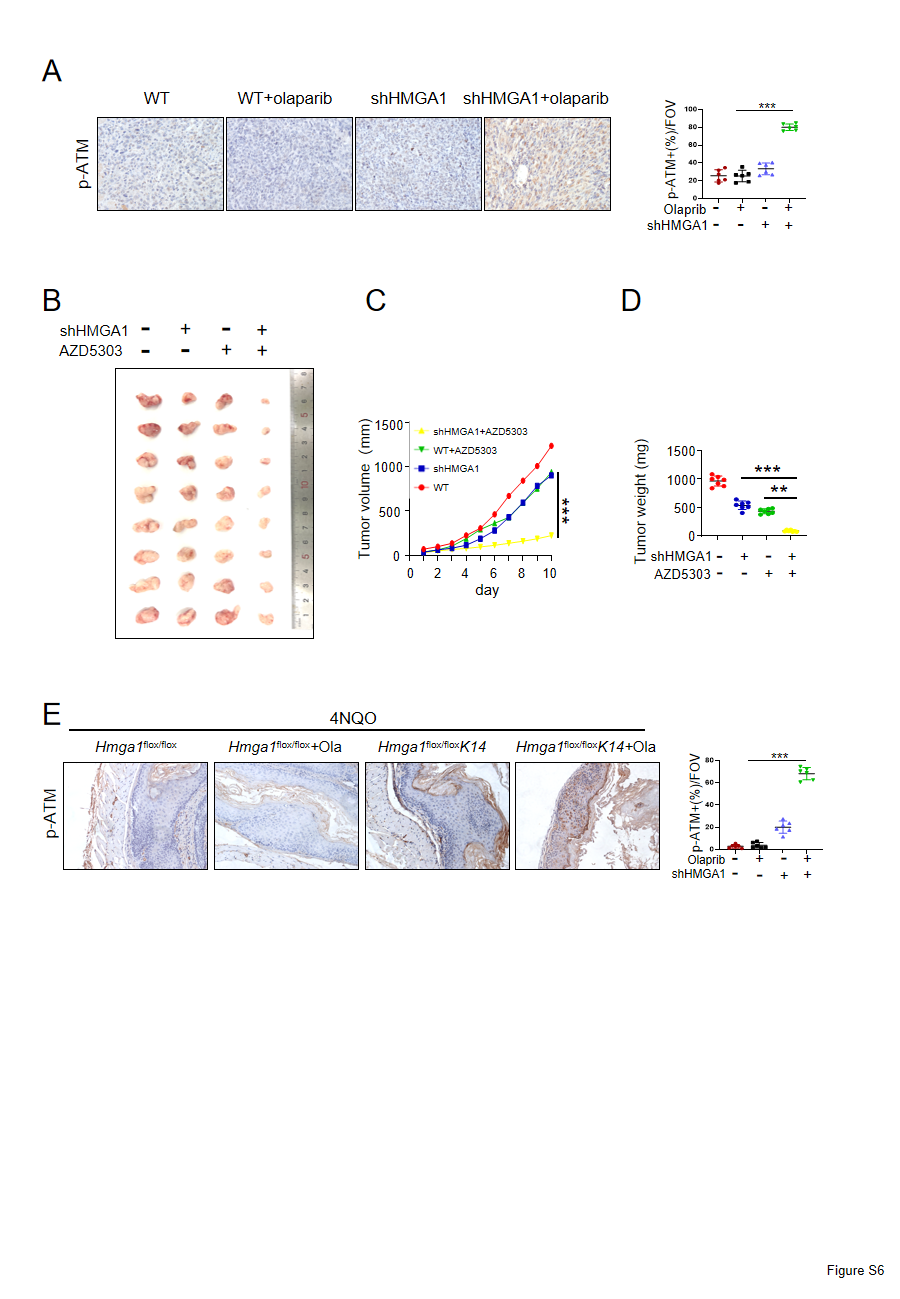

Supplement: Supplementary file 6 — Supporting information [file CTM2-14-e70111-s006.tif]

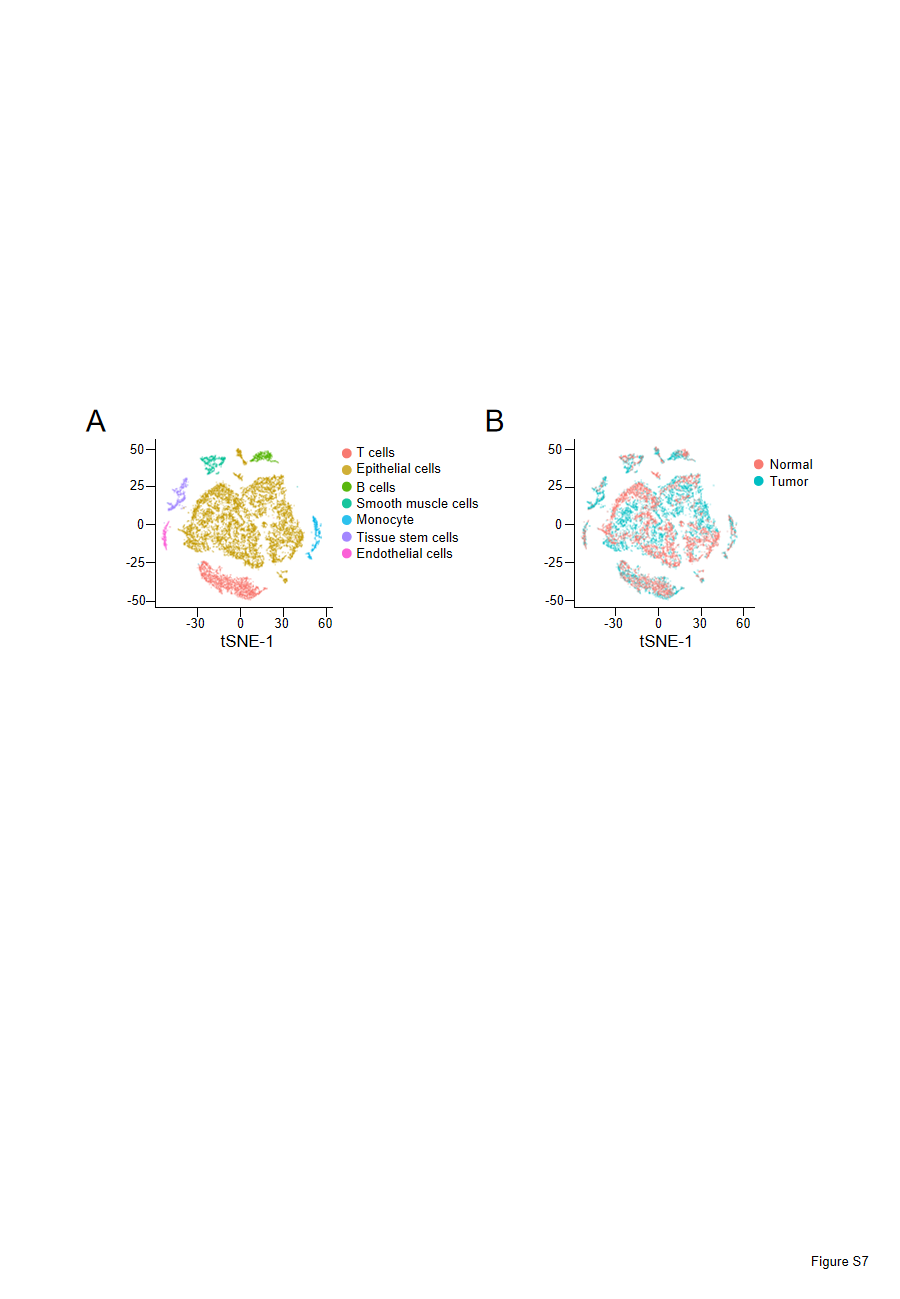

Supplement: Supplementary file 7 — Supporting information [file CTM2-14-e70111-s003.tif]
